# Metabolomics revealed the influence of breast cancer on lymphatic endothelial cell metabolism, metabolic crosstalk, and lymphangiogenic signaling in co-culture

**DOI:** 10.1038/s41598-020-76394-7

**Published:** 2020-12-04

**Authors:** Suehelay Acevedo-Acevedo, Douglas C. Millar, Aaron D. Simmons, Peter Favreau, Paulo F. Cobra, Melissa Skala, Sean P. Palecek

**Affiliations:** 1 Department of Biomedical Engineering, University of Wisconsin-Madison, Madison, WI 53706 USA; 2 Department of Chemical and Biological Engineering, University of Wisconsin-Madison, Madison, WI 53706 USA; 3 Morgridge Institute for Research, Madison, WI USA; 4 Department of Biochemistry, University of Wisconsin-Madison, Madison, WI 53706 USA

**Keywords:** Metabolomics, Breast cancer, Cancer metabolism, Cancer microenvironment

## Abstract

Breast cancer metastasis occurs via blood and lymphatic vessels. Breast cancer cells ‘educate’ lymphatic endothelial cells (LECs) to support tumor vascularization and growth. However, despite known metabolic alterations in breast cancer, it remains unclear how lymphatic endothelial cell metabolism is altered in the tumor microenvironment and its effect in lymphangiogenic signaling in LECs. We analyzed metabolites inside LECs in co-culture with MCF-7, MDA-MB-231, and SK-BR-3 breast cancer cell lines using $^1\hbox {H}$ nuclear magnetic resonance (NMR) metabolomics, Seahorse, and the spatial distribution of metabolic co-enzymes using optical redox ratio imaging to describe breast cancer-LEC metabolic crosstalk. LECs co-cultured with breast cancer cells exhibited cell-line dependent altered metabolic profiles, including significant changes in lactate concentration in breast cancer co-culture. Cell metabolic phenotype analysis using Seahorse showed LECs in co-culture exhibited reduced mitochondrial respiration, increased reliance on glycolysis and reduced metabolic flexibility. Optical redox ratio measurements revealed reduced NAD(P)H levels in LECs potentially due to increased NAD(P)H utilization to maintain redox homeostasis. $^{13}\hbox {C}$-labeled glucose experiments did not reveal lactate shuttling into LECs from breast cancer cells, yet showed other $^{13}\hbox {C}$ signals in LECs suggesting internalized metabolites and metabolic exchange between the two cell types. We also determined that breast cancer co-culture stimulated lymphangiogenic signaling in LECs, yet activation was not stimulated by lactate alone. Increased lymphangiogenic signaling suggests paracrine signaling between LECs and breast cancer cells which could have a pro-metastatic role.

## Introduction

Breast cancer is one of the most commonly diagnosed cancers among women worldwide^1,2^. Metastatic breast cancer has a 5-year survival of 25.9% compared to 98.6% for localized breast cancer^3^. The presence of blood and lymphatic vessels in tumors is vital to disease progression and metastasis^4–9^. Due to the rapid rate of tumor growth and vascularization, tumor blood vessels tend to be leaky^10,11^, which provides a conduit for cancer cells with metastatic potential to reach distant organs. Furthermore, lymphatic vessels are a favorable route of metastasis since primary tumors can express lymphangiogenic growth factors, vascular endothelial growth factor-C (VEGF-C) and VEGF-D, which promote tumor-associated lymphangiogenesis thus leading to invasion into the lymphatic vasculature, lymph node metastasis, and tumor dissemination^6,7,12,13^. Expression of these lymphangiogenic growth factors correlated with poor patient prognosis in certain human cancers such as invasive breast carcinoma, lung, prostate, colorectal, and thyroid cancer^12,14–16^. Lee *et al.* reported that metastatic tumor cells ‘educate’ lymphatic endothelial cells (LECs) to promote tumor growth by stimulating secretion of epidermal growth factor (EGF) and platelet-derived growth factor BB (PDGF-BB) in LECs conditioned by triple-negative breast cancer MDA-MB-231 cells^17^. A recent report by Ayuso *et al.* showed that breast cancer cells altered transcription of LECs which correlated with glucose permeability into the vasculature^18^.

An increasing number of studies have shed light into the metabolic environment within tumors and how stromal cells within the tumor microenvironment (TME) contribute to it. For example, cancer-associated fibroblasts (CAFs) isolated from basal-like breast cancers stimulated glucose uptake in breast cancer cells, while CAFs from less-aggressive breast cancer sub-types did not elicit the same response^19^. Whitaker-Menezes *et al.* reported that CAFs cultured with breast cancer cells expressed higher levels of monocarboxylate transporter 4 (MCT4) and exported more lactate thus stimulating monocarboxylate transporter 1 (MCT1) expression in breast cancer cells^20^. Furthermore, there have been recent advances in the development of protocols to allow in depth study of cell metabolism^21,22^.

Endothelial cells, including LECs, rely mainly on glycolysis to produce ATP given that they need to be able to proliferate in low oxygen environments, such as during angiogenesis^23,24^. Furthermore, tumor-associated endothelial cells are significantly more glycolytic than their healthy counterparts^25^. Additional studies into endothelial cell metabolism also revealed that lactate promotes angiogenesis in endothelial cells via activation of nuclear factor kappa-light-chain-enhancer of activated B cells (NF-$\kappa$B), interleukin 8 (IL-8), and hypoxia-inducible factor 1$\alpha$ (HIF-1$\alpha$) signaling^26–29^. This activation of angiogenesis in response to lactate has been shown to support tumor metastasis^30–32^. For these reasons, endothelial cell metabolism can be a powerful target to reduce tumor metastasis. A recent study showed that reduction of glycolysis in tumor-associated endothelial cells via partial inhibition of PFKFB3 resulted in a tighter endothelial barrier and reduced metastasis^25^.

In recent years, studies have described the role of lymphatic endothelial cells in the TME^18,23^. However, lymphatic endothelial cell metabolism in the TME has not been widely explored and additional research is needed to further understand cancer-endothelial cell interactions which comprise a key aspect of metastasis development. The relationship between lymphatic endothelial and breast cancer cell metabolism remains unclear. This article describes the metabolic changes occurring in LECs when cultured with breast cancer cells and if they influence lymphangiogenesis. Breast cancer co-culture was found to be associated with many metabolic alterations in LECs. LECs in co-culture exhibited reduced mitochondrial respiration, increased reliance on glycolysis and reduced metabolic flexibility according to Seahorse analysis. This corresponded to a quiescent endothelial cell metabolic phenotype which supported the reduced NAD(P)H levels detected by optical redox ratio measurements potentially due to increased NAD(P)H utilization to maintain redox homeostasis. Increased intracellular lactate was further studied to determine the state of lactate shuttling and utilization and its role in lymphangiogenesis. Our data showed that LECs upregulated lactate metabolism markers (LDHs and MCTs), did not import lactate originating from breast cancer cells but did uptake other metabolites from breast cancer cells. Interestingly, LECs co-cultured with breast cancer cells had reduced oxygen consumption rate (OCR and metabolic potential.) Furthermore, lactate alone was not sufficient to stimulate lymphangiogenic signaling in LECs, yet lymphangiogenic markers were increased in breast cancer co-culture, particularly for MDA-MB-231 co-culture, suggesting additional paracrine signaling driving this increase. Taken together, this study characterized the metabolic reprogramming LECs undergo in the presence of breast cancer cells and determined that lactate alone did not stimulate lymphangiogenesis in our co-culture system.

## Results

### Alterations in LEC metabolism were breast cancer cell sub-type dependent

**Figure 1:**
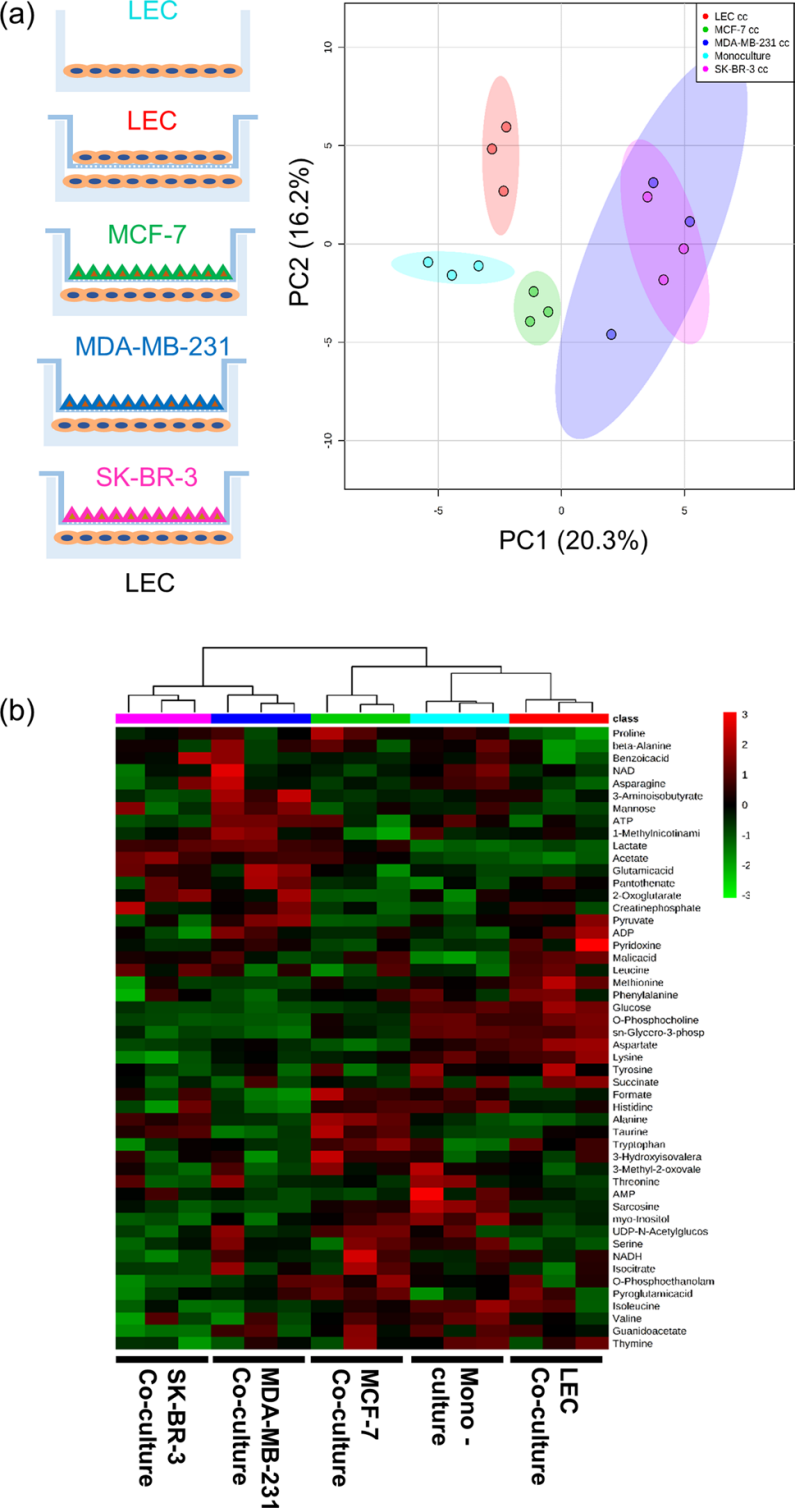
Global LEC metabolism is altered in co-culture with breast cancer cell lines. (**a**) PCA score plot depicts the clustering patterns from the metabolite concentration data. Shaded ellipses represent the 95% confidence interval region. (**b**) Hierarchical clustering heatmap shows distinct metabolic profiles between monoculture (MC; cyan, LECs cultured alone), LEC cc [LECs cultured on the top and bottom Transwell chambers (red, cell density control)], MCF-7 cc (green, LECs co-cultured with MCF-7 breast cancer cells), MDA-MB-231 cc (blue, LECs co-cultured with MDA-MB-231 cells), or SK-BR-3 cc (pink, LECs co-cultured with SK-BR-3 cells). For hierarchical clustering, Pearson’s correlation was used as a similarity measure and the clustering algorithm utilized was Ward’s linkage. Individual metabolites are shown in rows and individual samples are organized in the columns. Heatmap legend shows autoscaled values for relative metabolite concentrations. Data were analyzed using Metaboanalyst^33^. n = 3 independent biological replicates per condition.

To determine what changes occurred in LEC metabolism in the presence of breast cancer cells, LECs were cultured for four days in a reduced medium composed of endothelial basal media 2 (EBM2) with 5% fetal bovine serum (FBS) using a Transwell co-culture system. This time point was selected to allow cells to reach confluency and ensure metabolic measurements captured the response of LECs after being co-cultured with breast cancer cells for a significant amount of time. The medium formulation allowed us to measure metabolic changes due to co-culture instead of stimulation from additional growth factors supplemented in the medium. The following conditions were analyzed: intracellular metabolites from LECs in monoculture (MC); LEC co-culture (cc, cell density control); MCF-7 cc; MDA-MB-231 cc; and SK-BR-3 cc (Fig. 1a).

First, a principal component analysis (PCA) was performed on the quantified metabolite data and revealed global differences in LEC metabolism upon co-culture with breast cancer cells (Fig. 1a). There was no overlap between the MC and LEC cc clusters, suggesting there were metabolic changes differentiating these two conditions, likely resulting from differences in cell number. Therefore, the LEC cc condition was used as control for all further experiments given that the total cell density matched the LEC-cancer cell co-culture conditions more closely. MCF-7 cc samples clustered separately from the other 4 sample groups indicating there were unique metabolic changes influenced by co-culture with MCF-7 cells. Interestingly, MDA-MB-231 cc and SK-BR-3 cc samples clustered together indicating that the metabolic changes occurring in the LECs cultured with the 2 breast cancer cell lines were similar.

The hierarchical clustering heatmap supported the clustering patterns obtained by PCA (Fig. 1b). The metabolic profiles shown in the heatmap further highlighted relative changes in individual metabolites which contributed to the metabolic differences between sample groups. The PCA and heatmap both indicated that LEC metabolism was affected by the presence of breast cancer cells and these metabolic changes in LECs were breast cancer cell line dependent.

**Figure 2:**
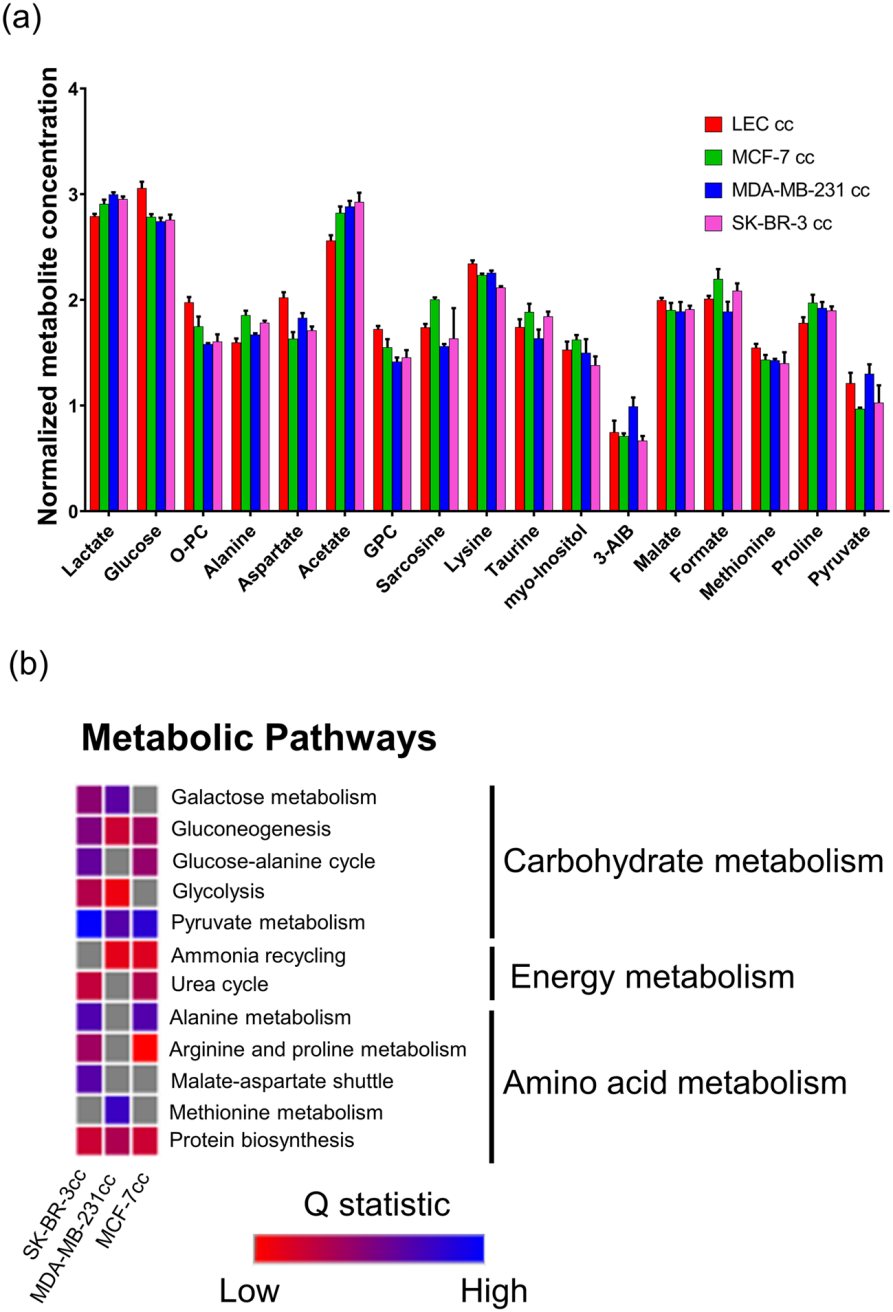
Multiple metabolites and metabolic pathways are altered in LECs co-cultured with breast cancer. (**a**) The top 17 significantly altered metabolites in LECs determined by one-way ANOVA and Tukey’s HSD post-hoc analysis (performed using Metaboanalyst^33^); FDR < 0.05 for all plotted metabolites. Error bars denote standard deviation; n = 3 independent biological replicates. (**b**) Metabolite set enrichment analysis (MSEA) was performed comparing LECs co-cultured with breast cancer compared to LEC cc control. Colored squares denote the Q statistic value which is an enrichment measure generated by MSEA and have an FDR < 0.05. Higher Q statistic values indicate higher pathway enrichment. Grey squares represent pathways that were not significantly enriched (FDR > 0.05). cc stands for co-culture. Analysis was performed using Metaboanalyst^33^ and the heatmap was generated using Morpheus software.

To identify significantly changing metabolites, one-way analysis of variance (ANOVA) with Tukey’s honest significant difference (HSD) post-hoc analysis was performed on the metabolite concentration data. This revealed 14 significantly altered metabolites between LEC cc and breast cancer co-culture conditions (false discovery rate [FDR] < 0.05; Fig. 2a). The altered metabolites mainly included organic acids, osmolytes, and amino acids. Eight metabolites were significantly different in co-culture with breast cancer cells compared to LEC cc control: lactate, glucose, o-phosphocholine (O-PC), aspartate, acetate, sn-glycero-phosphocholine (GPC), lysine, and methionine. Several metabolites significantly changed for LECs co-cultured with two out of the three breast cancer cell lines compared to LEC cc. For example, alanine was significantly increased in MCF-7 cc and SK-BR-3 cc conditions compared to LEC cc, and MDA-MB-231 cc. Taurine, formate, and proline were significantly different between LEC cc and MCF-7 cc but not for the other two breast cancer co-culture conditions. 3-aminoisobutyrate (3-AIB) was significantly increased for MDA-MB-231 cc relative to all other conditions analyzed.

For quantitative metabolite set enrichment analysis (MSEA) measurements, we obtained a list of enriched pathways based on all metabolites quantified. Thirteen metabolic pathways within this list were significantly different between LEC cc and breast cancer co-culture (Fig. 2b) and they corresponded to carbohydrate, energy waste management, and amino acid metabolism. Gluconeogenesis, pyruvate metabolism, and protein biosynthesis pathways were significantly different for all 3 breast cancer co-culture conditions relative to LEC cc. Glycolysis was significantly enriched for SK-BR-3 cc and MDA-MB-231 cc compared to LEC cc but not for MCF-7 cc. Furthermore, four metabolites involved in glycolysis and pyruvate metabolism (lactate, glucose, acetate, and pyruvate) were significantly different between LEC cc and breast cancer co-cultures, which contribute to the enrichment of these pathways. For all 3 breast cancer co-cultures, glucose levels were significantly reduced, and lactate and acetate levels were significantly increased. Meanwhile, pyruvate levels were significantly increased for MDA-MB-231 cc compared to MCF-7 cc and SK-BR-3 cc. Quantified metabolites involved in the tricarboxylic acid (TCA) cycle or the mitochondrial electron transport chain pathways did not reveal significant changes in metabolite concentrations between LEC cc and breast cancer co-culture (Fig. 2b). In conclusion, breast cancer co-culture stimulated significant changes in metabolite concentrations and pathways in LECs in a breast cancer cell-type dependent manner.

### LECs in co-culture may rely on glycolysis and have reduced ability to adapt to an energetic demand

**Figure 3:**
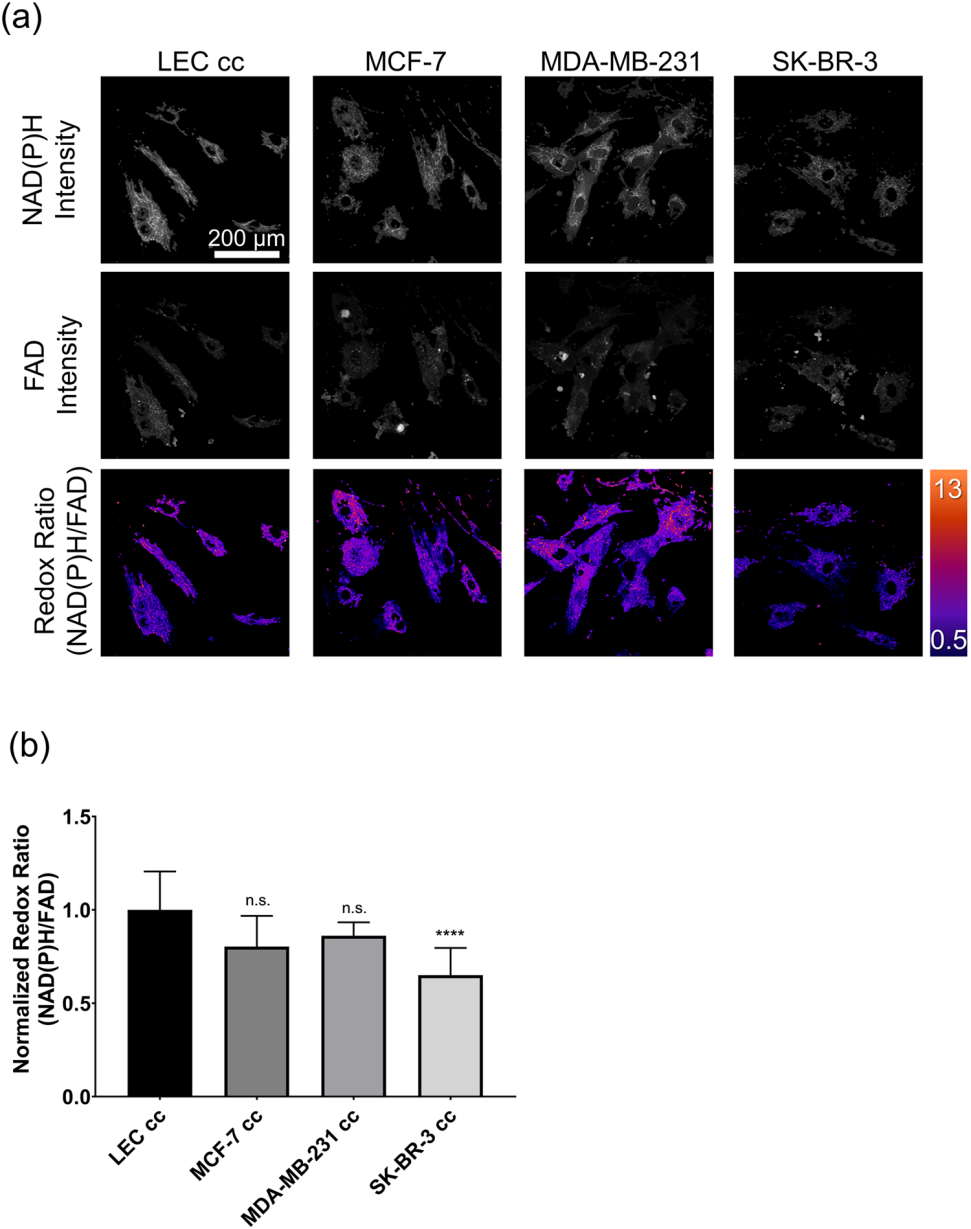
Images and optical redox ratio results from LEC co-culture experiments. (**a**) Representative images of NAD(P)H, FAD, and the optical redox ratio in co-cultures of LECs and the indicated breast cancer cell lines. (**b**) Quantified optical redox ratio for each co-culture. The redox ratio was normalized to the average across all LEC co-culture images. Error bars represent standard deviation. n = 6–16 fields of view. Statistics were calculated using a Kruskal–Wallis test (GraphPad Prism 7.04). ****$p<0.0001$. n.s. = not significant (*p* value > 0.05).

Since endothelial cells mainly rely on glycolysis, which was enriched in MSEA (Fig. 2b), we performed functional measurements of cell metabolism to confirm MSEA results. First, we performed optical metabolic imaging (OMI), which uses fluorescence from metabolic co-enzymes nicotinamide adenine dinucleotide phosphate (NAD(P)H) and flavin adenine dinucleotide (FAD) and is a nondestructive technique to study metabolism and provide a spatial map of electron donors and acceptors within single cells. OMI has been demonstrated to accurately quantify metabolic shifts in *in vitro* and *in vivo* cancer models^34–36^. NAD(P)H and FAD intensities were acquired 4 days after co-culture initiation and the redox ratio (NAD(P)H intensity/ FAD intensity) was quantified for each co-culture condition. Traditionally, a high redox ratio indicates increased glycolysis while a low redox ratio can be associated with increased oxidative phosphorylation. Representative images in Fig. 3 show the individual NAD(P)H and FAD images as well as representative optical redox ratio images. There was no change in redox ratio for MDA-MB-231 cc and MCF-7 cc compared to LEC cc. SK-BR-3 cc had a significantly reduced redox ratio. These results would suggest that LECs in co-culture with SK-BR-3s have reduced glycolysis in comparison to LEC cc, MDA-MB-231 cc and MCF-7 cc conditions.

**Figure 4:**
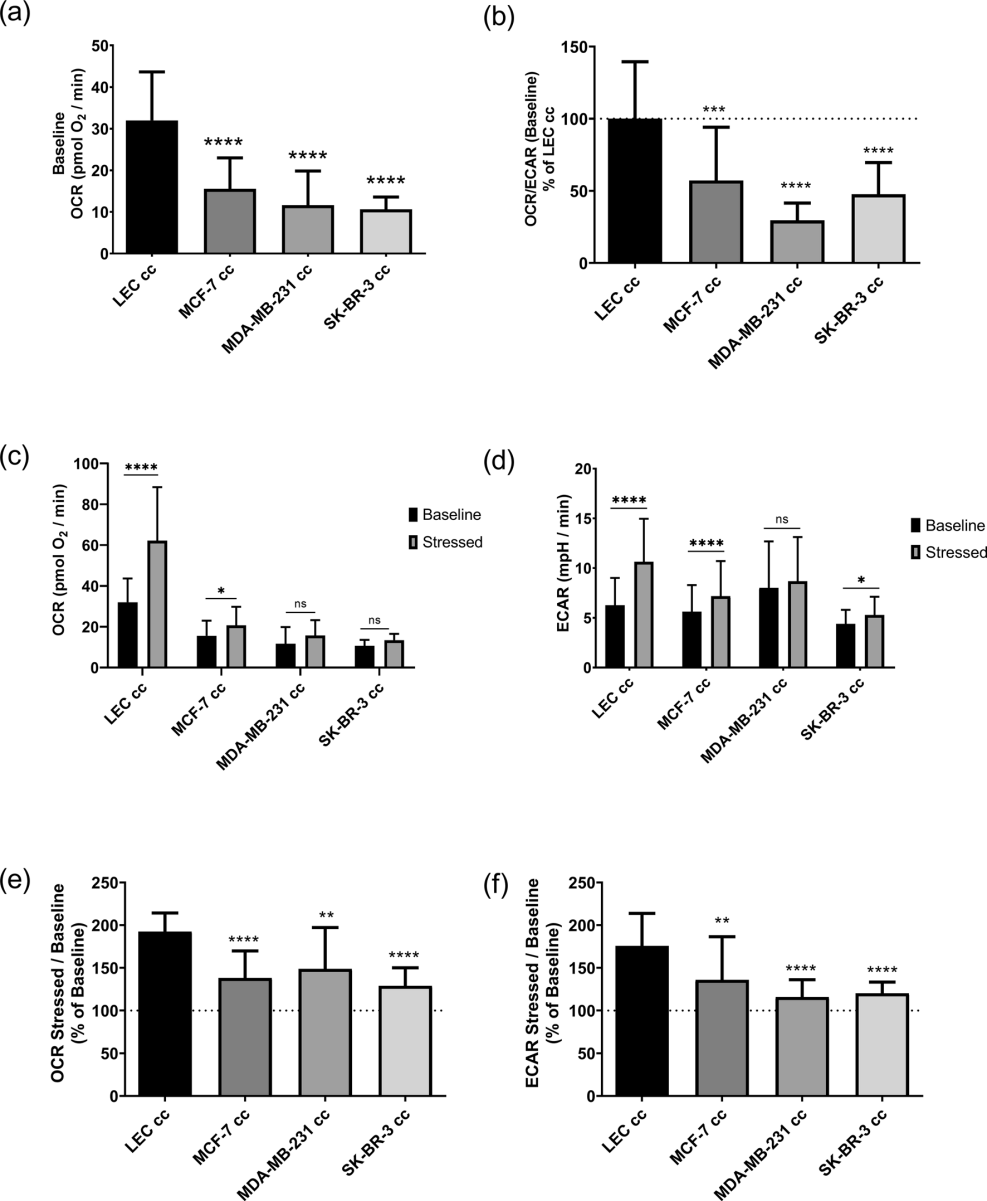
LECs co-cultured with breast cancer cells had reduced mitochondrial respiration and increased reliance on glycolysis. (**a**) Baseline oxygen consumption rate (OCR) measurements of LECs co-cultured (cc) with breast cancer cells. (**b**) Baseline OCR: ECAR ratio graphed as % of LEC cc. (**c**) OCR and (**d**) ECAR were measured at baseline and after oligomycin and FCCP addition (stressed condition). The ratio of stressed to baseline response was calculated for (**e**) OCR and (**f**) ECAR measurements. Statistics were calculated using one-way ANOVA with Sidak’s multiple comparisons test (GraphPad Prism 8.03). *p value < 0.05; **p value < 0.005; ***p value < 0.0005; ****p value < 0.0001; n.s. = not significant (*p* value > 0.05). n = 12–17 biological replicates. Error bars represent standard deviation.

To complement redox ratio measurements and determine if LECs, indeed, had reduced glycolysis, we measured the oxygen consumption rate (OCR) and extracellular acidification rate (ECAR) of LECs co-cultured with breast cancer cells (Fig. 4). At baseline, LECs co-cultured with breast cancer cells had significantly reduced OCR (Fig. 4a) values compared to LEC cc. There was no change in ECAR values compared to LEC cc (Fig. S3a). The OCR: ECAR ratio was significantly reduced for LECs co-cultured with breast cancer cells suggesting an increased dependence on glycolysis compared to LEC cc (Fig. 4b).

In order to evaluate the metabolic response of LECs under stress, we induced metabolic stress in LECs by introducing an increase in energy demand by adding oligomycin and FCCP to the cells after baseline measurements. This stressed condition was then compared to the baseline OCR and ECAR measurements. The stressed OCR values for MCF-7 cc were significantly higher but not for MDA-MB-231 cc and SK-BR-3 cc conditions (Fig. 4c). For ECAR measurements, MCF-7 cc and SK-BR-3 cc conditions had significantly higher values in the presence of stressor compounds whereas MDA-MB-231 cc samples did not (Fig. 4d). Furthermore, the stressed: baseline ratio was calculated to determine the metabolic potential of LECs co-cultured with breast cancer cells. All 3 co-culture conditions showed significantly reduced stressed: baseline ratio compared to LEC cc indicating that breast cancer co-culture reduced the ability of LECs to adapt to an increase in energy demand (Fig. 4e–f). Given that lactate was significantly increased inside LECs, we measured the basal and stressed OCR and ECAR values of LECs cultured in medium supplemented with 10 mM lactate (Fig. S9). Interestingly, lactate supplementation reduced basal OCR levels (Fig. S9a) yet did not affect the ability of LECs to respond to an increase in energy demand (Fig. S9d–g). Overall, LECs in co-culture with breast cancer likely shifted to a more quiescent metabolic phenotype^23,37,38^ in co-culture evidenced by their reliance on glycolysis, reduced OCR, and reduced metabolic potential compared to LEC cc (Fig. S3b).

### Breast cancer co-culture significantly impacted lactate metabolism markers in LECs

Since lactate was significantly increased in LECs co-cultured with breast cancer cells (Fig. 2a) and lactate has been shown to have a role in vascular endothelial cell angiogenesis, we tested if breast cancer cell conditioned medium was sufficient to increase lactate levels in LECs (Fig. S4a). Lactate was significantly increased inside LECs cultured in breast cancer conditioned medium compared to control medium by a factor of 0.62–1.2. To determine whether the elevated levels of intracellular lactate in LECs co-cultured with breast cancer cells corresponded to higher levels of secreted lactate, we quantified lactate concentration in the media (Fig. S4b). Lactate concentrations in MCF-7 cc and SK-BR-3 cc media were similar to the concentration measured in LEC cc medium. However, lactate levels in MDA-MB-231 cc medium conditions were significantly increased by 0.6 compared to LEC cc medium.

**Figure 5:**
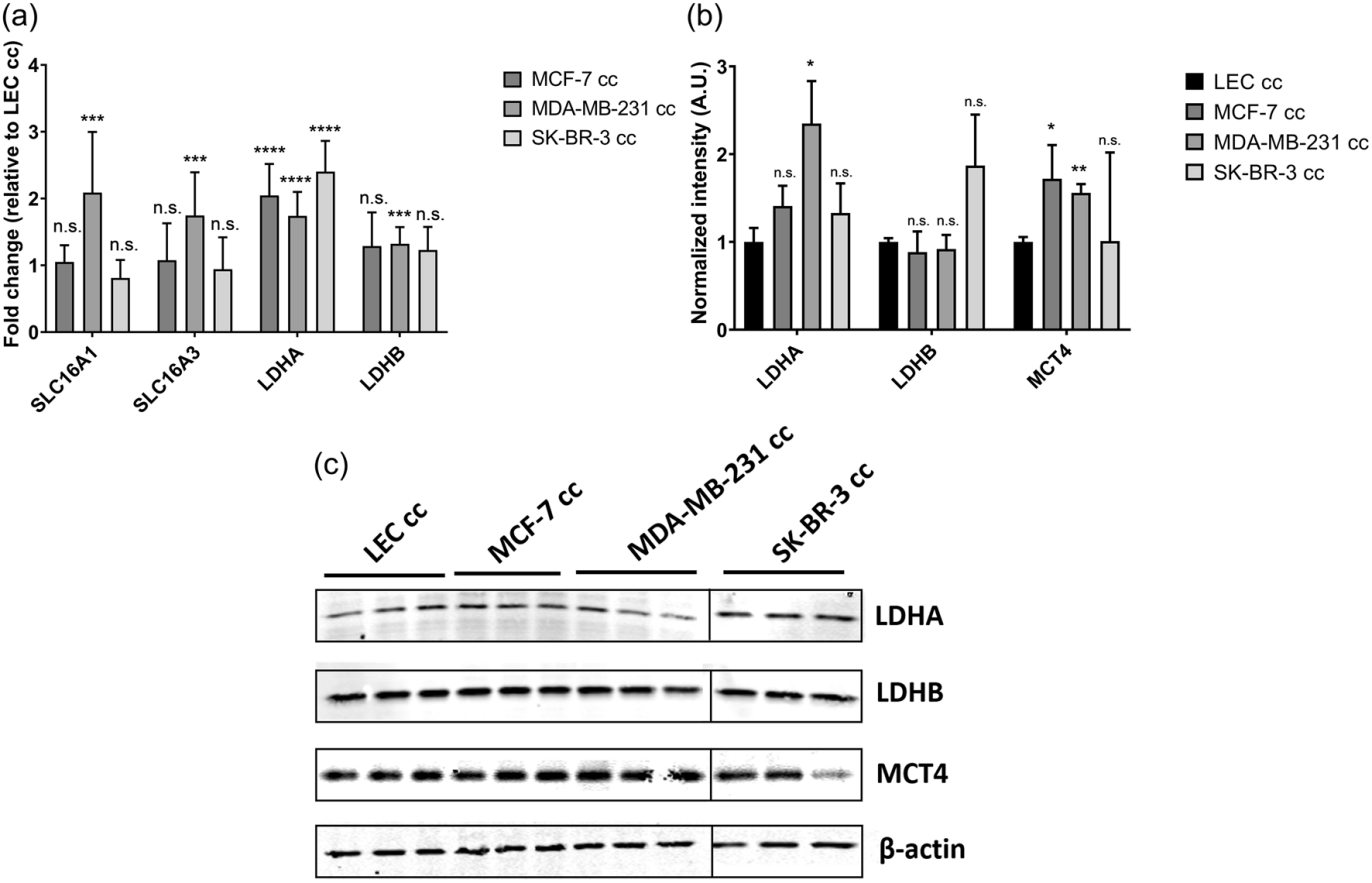
Genes and proteins associated with lactate metabolism and transport were quantified for LECs co-cultured with the indicated breast cancer cell lines. (**a**) qRT-PCR analysis of *SLC16A1, SLC16A3, LDHA, and LDHB* gene expression in LECs was measured 4 days after co-culture initiation. Gene expression values were normalized to glyceraldehyde 3-phosphate dehydrogenase *(GAPDH)* expression and fold change calculations were normalized to LEC cc. n = 9 biological replicates; 3 independent experiments. (**b**) Quantification of LDHA, LDHB, and MCT4 protein expression in LECs 4 days after co-culture initiation. Band intensity values were normalized to $\beta$-actin expression. (**c**) Representative western blot images of LECs co-cultured with breast cancer cells. The SK-BR-3 cc blot was run in a separate gel with its own LEC cc control; indicated here by a black vertical line. Full blots are in Fig. S2. For display images, the background was subtracted, and the signal was sharpened using ImageJ. n = 3 biological replicates. Statistical significance compared to LEC cc was calculated using a t-test with two-step Benjamini, Krieger and Yekutieli procedure (GraphPad Prism 7.04). *p value < 0.05; **p value < 0.01; ***p value < 0.005; ****p value < 0.00005. n.s. = not significant (*p* value > 0.05). Error bars represent standard deviation.

We further investigated the role lactate played in LECs given the extensive literature describing the role of lactate in vascular endothelial cell metabolism and angiogenesis. Quantitative reverse transcription-polymerase chain reaction (RT-PCR) was performed to evaluate the expression of genes involved in lactate metabolism and transport; specifically, *Solute Carrier Family 16 Member 1 (SLC16A1)* which encodes for MCT1, *Solute Carrier Family 16 Member 3 (SLC16A3)* which encodes for MCT4, *lactate dehydrogenase A (LDHA)*, and *LDHB* (Fig. 5a). *LDHA* and *LDHB* encode two isoforms of the LDH enzyme which catalyzes the interconversion of pyruvate to lactate. *LDHA* gene expression was significantly upregulated in all breast cancer co-culture conditions relative to LEC cc (Fig. 5a). LDHA protein levels were increased for all breast cancer co-culture conditions matching qRT-PCR data (Fig. 5). *LDHB* gene expression was significantly upregulated for MDA-MB-231 cc samples, yet there was no change in protein levels in MDA-MB-231 cc compared to LEC cc. There was no change in LDHB protein levels in MCF-7 cc compared to LEC cc, and LDHB protein expression increased in SK-BR-3 cc compared to LEC cc, yet this increase was not statistically significant (Fig. 5b). We showed that lactate metabolism markers were significantly affected by breast cancer co-culture correlating with increased lactate levels observed.

MCT1 and MCT4 have been associated with lactate import and export, respectively, via the vascular endothelial lactate shuttle model^39^. *SLC16A1* and *SLC16A3* expression levels were significantly upregulated only for MDA-MB-231 cc compared to LEC cc (Fig. 5a) suggesting increased lactate shuttling into and out of the cells. Increased lactate shuttling would also be consistent with the higher levels of lactate present in the MDA-MB-231 cc medium (Fig. S4a). Relative protein levels of MCT4 were quantified by western blot (Fig. 5). MCT1 was not quantified due to the lack of availability of a specific antibody. MCT4 protein levels were significantly higher for MCF-7 cc and MDA-MB-231 cc compared to LEC cc samples but not for SK-BR-3 cc. However, there was no change in *SLC16A3* gene expression in MCF-7 cc compared to LEC cc.

**Figure 6:**
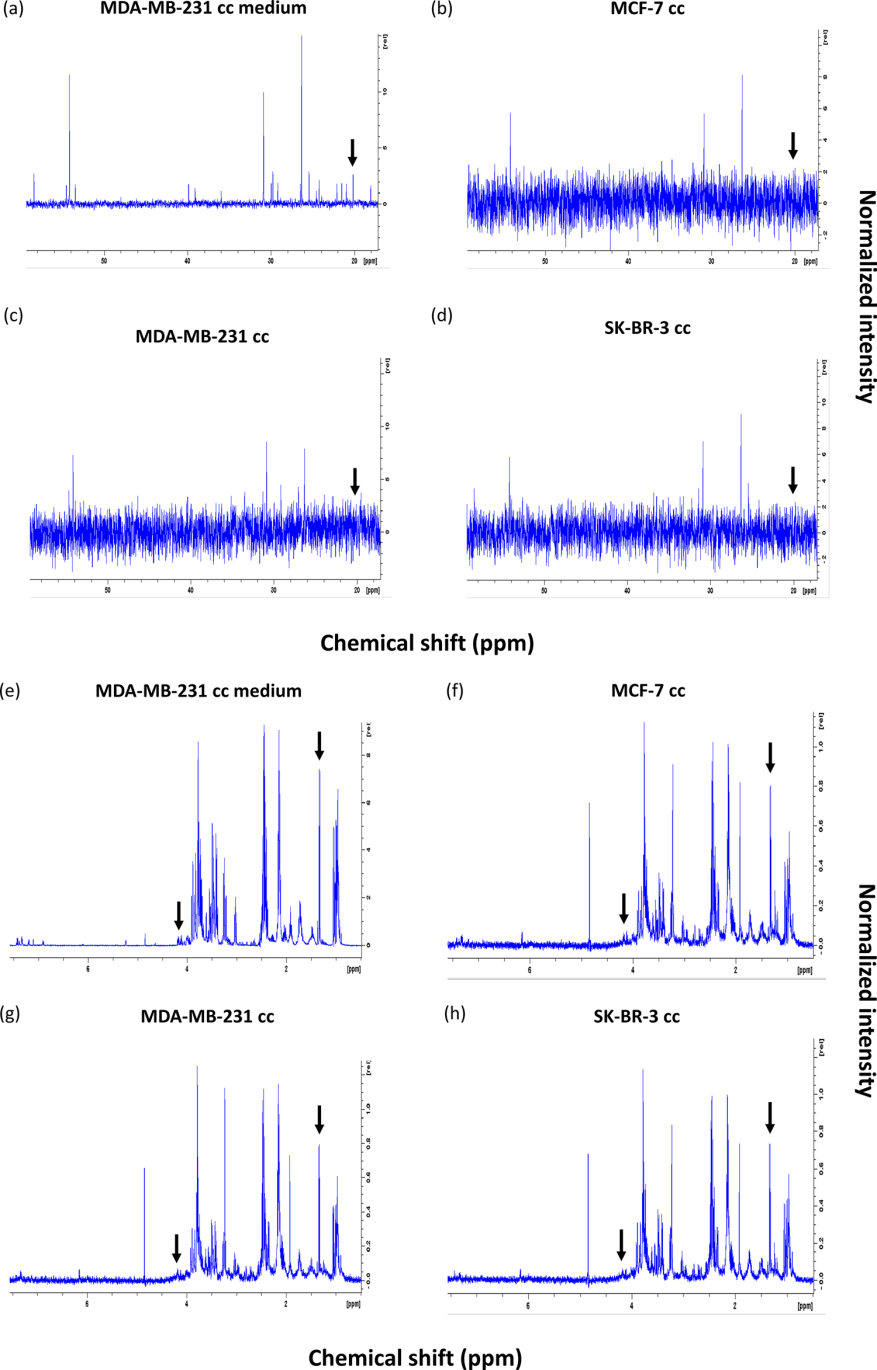
LECs imported metabolites secreted by breast cancer cells. (**a**–**d**) Representative $^{13}\hbox {C}$ NMR spectra of medium from LECs co-cultured with MDA-MB-231 (**a**), LECs co-cultured with MCF-7 (**b**), MDA-MB-231 (**c**), SK-BR-3 (**d**) cells. (**e**–**h**) Representative $^{1}\hbox {H}$ NMR spectra of medium from LECs co-cultured with MDA-MB-231 (**e**), LECs co-cultured with MCF-7 (**f**), MDA-MB-231 (**g**), and SK-BR-3 (**h**) cells. Black arrows point to lactate peak(s) in the spectrum. Normalized intensity axis for medium sample (**a**) is larger compared to normalized intensity axes in breast cancer co-culture spectra due to higher metabolite concentration in the medium.

To determine if lactate was shuttled from breast cancer cells into LECs, breast cancer cells were cultured in medium containing $^{13}\hbox {C}$-labeled glucose for 24 h. Subsequently, breast cancer cells and LECs were co-cultured in medium free of $^{13}\hbox {C}$-labeled glucose for 6 h. LECs were harvested and analyzed using $^{13}\hbox {C}$ NMR. A reference $^{13}\hbox {C}$-lactate NMR spectrum from the Biological Magnetic Resonance Bank (BMRB) database (BMRB ID: bmse000208) was used to determine if the corresponding lactate peak was present within the $^{13}\hbox {C}$ NMR spectra. There was no detectable peak where $^{13}\hbox {C}$-lactate should be (Fig. 6b–d, black arrow). $^{1}\hbox {H}$ NMR spectra of these samples revealed that unlabeled lactate was present inside the LECs (Fig. 6e–f) and MDA-MB-231 cc medium analysis showed $^{13}\hbox {C}$-lactate present in the medium (Fig. 6a). 2D $^{1}\hbox {H}$–$^{13}\hbox {C}$ heteronuclear single quantum coherence (HSQC) NMR spectra confirmed that $^{13}\hbox {C}$-lactate was not present inside LECs. Nevertheless, both 1D $^{13}\hbox {C}$ NMR and 2D $^{1}\hbox {H}$–$^{13}\hbox {C}$ HSQC NMR spectra revealed $^{13}\hbox {C}$ signals that show LECs do import metabolites secreted by breast cancer cells (Fig. 6 and S5). Additional analysis is needed, however, to identify these metabolites and is the subject of follow-up work.

LECs were also cultured in high lactate conditions to gain further insight into the source of the high intracellular lactate levels and further explore the role of lactate in these cells. LECs cultured in media spiked with 10 mM sodium lactate were analyzed by $^1\hbox {H}$ NMR. PCA and hierarchical clustering revealed global metabolic differences between LECs cultured in control media and lactate-spiked media (Fig. S6a–b). Four metabolites, including lactate, contributed significantly to the metabolic differences (Fig. S6c) and the enriched metabolic pathways for LECs cultured in lactate-spiked media included pyruvate metabolism and the Warburg effect (Fig. S7). Redox ratio measurements showed a significantly higher redox ratio for LECs cultured in 10 mM lactate (Fig. S8), possibly as a result of increased NAD(P)H production due to increased fatty acid oxidation. There was no change in LDHA, LDHB and MCT4 protein levels in lactate-spiked LECs relative to control LECs (Fig. S11). Yet, mRNA levels of *SLC16A1, SLC16A3, LDHA*, and *LDHB* were significantly downregulated in lactate-spiked samples (Fig. S8).

### Breast cancer co-culture promoted upregulation of lymphangiogenesis markers in LECs

**Figure 7:**
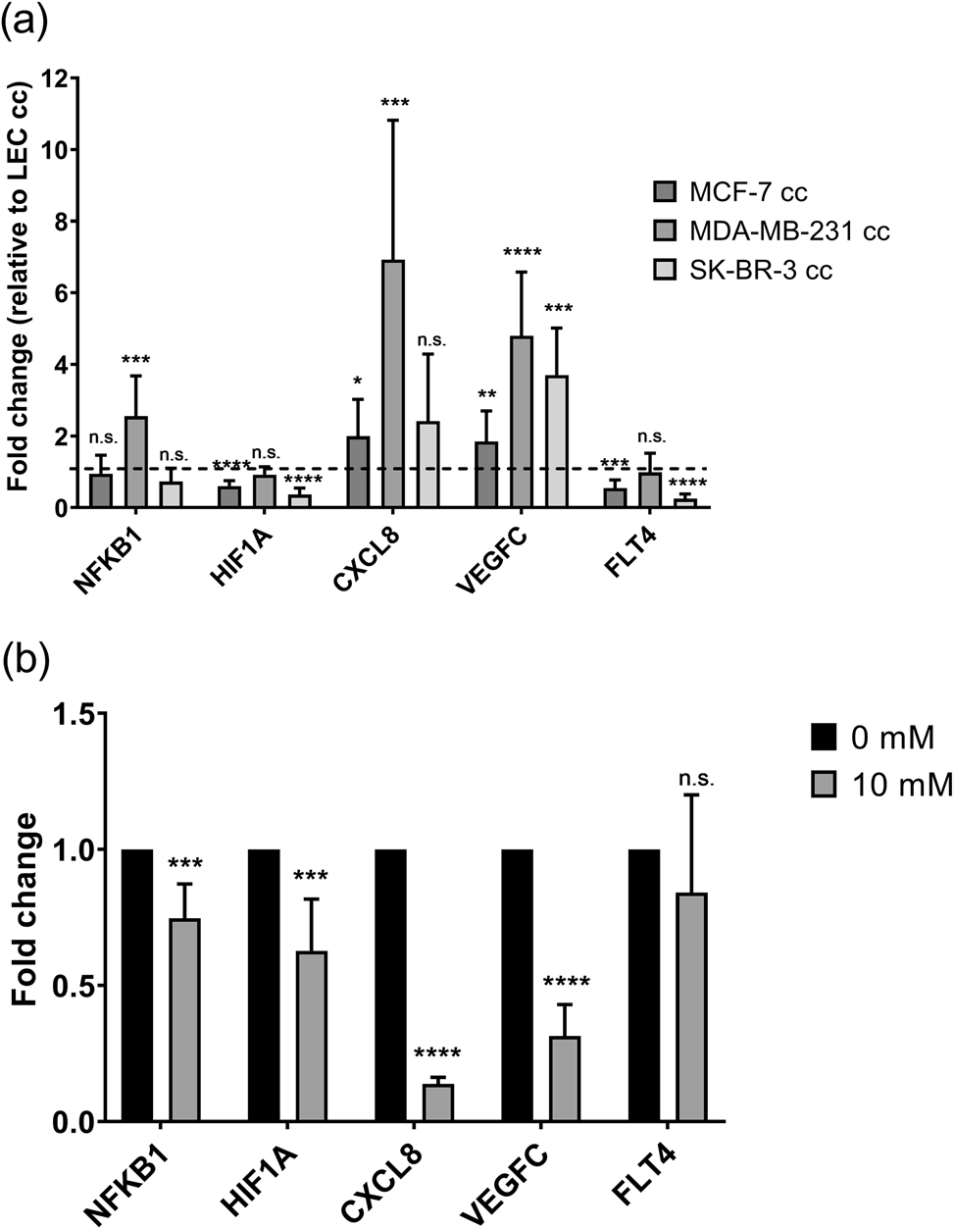
Co-culture with invasive breast cancer cells promoted significant upregulation of lymphangiogenic signaling. qRT-PCR of LECs co-cultured with the indicated breast cancer cells (**a**) was performed 4 days after co-culture initiation. (**b**) qRT-PCR of lymphangiogenesis markers in LECs cultured in high lactate medium was assayed after 4 days of culture. Genes analyzed were *NFKB1*, $HIF1A$, *CXCL8*, *VEGFC*, and *FLT4*. Gene expression was normalized by *GAPDH* expression and fold change was calculated compared to LEC cc or 0 mM lactate, respectively. n = 8–9 samples per condition; 3 independent experiments. Statistics were calculated using a t-test with two-step Benjamini, Krieger and Yekutieli procedure (GraphPad Prism 7.04). *p value < 0.05; **p value < 0.01; ***p value < 0.001; ****p value < 0.00001. n.s. = not significant (*p* value > 0.05). Error bars represent standard deviation.

Increased lactate inside endothelial cells has been shown to stimulate angiogenic signaling, specifically increased gene expression and activity of HIF1$\alpha$, NF-$\kappa$B, and IL-8 which contribute to an increase in tumor metastasis^27–29,40^. We measured mRNA levels of *NFKB1, *$HIF1A$, *CXCL8, VEGFC*, and *fms related tyrosine kinase 4 (FLT4)* in LECs co-cultured with breast cancer (Fig. 7a). VEGF-C and FLT4 are lymphatic specific VEGF receptor and ligand. LECs co-cultured with breast cancer cells upregulated some lymphangiogenic markers. *VEGFC* was significantly upregulated in LECs co-cultured with breast cancer compared to control. *CXCL8* was significantly upregulated for MCF-7 cc and MDA-MB-231 cc, while *NFKB1* was significantly upregulated only for MDA-MB-231 cc. Therefore, breast cancer co-culture did stimulate some upregulation of lymphangiogenesis-associated genes. There was also significant downregulation of gene expression of $HIF1A$ and *FLT4* for some breast cancer co-culture conditions.

Given the upregulation of certain lymphangiogenic genes, we performed a cord formation assay on LECs in co-culture to evaluate *in vitro* tubulogenic potential (Fig. S12). There were no differences in cord, branching and junction length between LEC cc and breast cancer cc. Subsequently, LECs were cultured in 10 mM lactate to determine if increased lactate alone was enough to stimulate lymphangiogenesis. LECs cultured in 10 mM lactate had significant downregulation of *NFKB1,* $\it HIF1A$, *CXCL8* and *VEGFC* (Fig. 7b). There was no significant change in *FLT4* expression for LECs compared to control. In conclusion, we determined that breast cancer co-culture stimulated lymphangiogenic signaling in LECs, yet activation was not stimulated by lactate alone.

## Discussion

The impact of breast cancer sub-types on the metabolism of LECs *in vitro* and their relation to lymphangiogenesis was investigated. These results provide an initial indicator of how LECs and breast cancer cells interact metabolically in the TME. Our results showed that LEC metabolism is influenced by breast cancer co-culture in a cell line-dependent manner (Fig. 1). Both highly invasive breast cancer cell lines clustered together in the PCA plot and could indicate that LEC metabolism is influenced differently by metastatic and non-metastatic cell lines; although, a much larger number of lines would need to be studied to draw this conclusion. Furthermore, 17 metabolites were significantly altered and 12 metabolic pathways, including glycolysis and pyruvate metabolism, were enriched in LECs co-cultured with breast cancer cell lines. Lactate levels were significantly increased in LECs co-cultured with breast cancer cells, which correlated to upregulation of lactate metabolism enzymes and transporters, particularly for LECs co-cultured with MDA-MB-231 cells. The Transwell system utilized for co-culture allowed soluble factor communication between breast cancer cells and LECs, and enabled us to easily harvest the metabolites from LECs using a mechanical dissociation method to minimize influencing the metabolic state of the cells. This system, however, did not allow direct cell contact. We cannot discount that the observed effects could be dependent on media formulation so further *in vivo* validation is the subject of follow-up work.

It is known that the endothelium is highly glycolytic compared to other tissues^41^. These changes in metabolite levels and pathways suggest that breast cancer co-culture affected the rate of glycolysis in LECs. This may explain the significantly higher intracellular lactate concentrations in LECs (Fig. S4a). Furthermore, LECs co-cultured with breast cancer cells had significantly lower OCR:ECAR ratio suggesting an increased dependence on glycolysis (Fig. 4b). This was due, however, to a significant reduction in OCR rather than an increase in ECAR. Moreover, a consequence of mitochondrial respiration is production of reactive oxygen species (ROS)^42^. Therefore, reduced OCR in LECs co-cultured with breast cancer cells could be a protective mechanism to reduce ROS inside LECs.

Interestingly, when LECs were challenged with an increase in energy demand by addition of mitochondrial stressor compounds (oligomycin and FCCP), the OCR and ECAR levels of LECs co-cultured with breast cancer cells did not change significantly from basal levels (Fig. 4c,d) with the exception of MCF-7 cc samples which had a reduced response to the increased energy demand compared to LEC cc. SK-BR-3 cc increased ECAR when stressed, suggesting an increase in glycolysis. Nevertheless, this response was reduced compared to LEC cc. This challenge corresponded with a significant reduction in the metabolic potential of LECs when co-cultured with breast cancer cells (Fig. 4). This would indicate that the ability of the LECs to respond to increases in energy demand was significantly reduced. For LECs cultured with 10 mM lactate, the ability of these cells to respond to an increase in energy demand was not affected by high lactate in the medium suggesting that this reduction in metabolic flexibility was not due solely to increased lactate levels in co-culture.

Optical redox ratio measurements seemed to contrast with Seahorse measurements since reduced redox ratio (Fig. 3) has been associated with increased oxidative phosphorylation while Seahorse analysis revealed significantly reduced OCR (Fig. 4a) which indicates reduced oxidative phosphorylation. Fatty acid oxidation has been shown to be important for lymphangiogenesis^43^ where it is utilized to produce Acetyl-CoA from fatty acids which generates NAD(P)H. One potential explanation for the results would be that LECs may be producing NAD(P)H via the TCA cycle and fatty acid oxidation. This NAD(P)H is then utilized by vasculoprotective proteins such as eNOS (NOS3), prostaglandin G/H synthase 1 (PTGS1), and glutaredoxin (GRX)^37^ to regulate redox homeostasis, thus leading to the reduced redox ratio seen particularly for SK-BR-3 cc. Also, the lower optical redox ratio in SK-BR-3 cc samples (Fig. 3) was consistent with lower NADH levels measured in SK-BR-3 cc NMR spectra compared to LEC cc (Fig. 1b) which would be consistent with increased utilization. This would lead to a more quiescent endothelial cell phenotype which we also observed for LECs in the Seahorse cell phenotype analysis (Fig. S3b).

Gene and protein expression of enzymes responsible for metabolizing lactate were increased in LECs co-cultured with breast cancer (Fig. 5). Considering transcription and translation are highly regulated processes with many intermediate steps, it is not entirely surprising to observe differences in mRNA levels inside LECs compared to protein levels. We showed that LECs co-cultured with MDA-MB-231 cells had significantly increased lactate metabolism genes. This implied increased lactate metabolism and potential lactate export for this particular condition given that the lactate concentration in the medium was significantly higher upon co-culture (Fig. S4b). Previous studies reported increased *SLC16A1* gene expression as well as high intracellular lactate in human umbilical vein endothelial cells (HUVECs), suggesting increased lactate shuttling into HUVECs^27^. However, LEC gene expression data of MCF-7 cc and SK-BR-3 cc suggested import and export of lactate between breast cancer cells and LECs could occur at similar rates since *SLC16A1 and SLC16A3* expression were similar. These results would suggest the possibility that the increased lactate in the LECs was mainly produced intracellularly instead of uptake of cancer cell-secreted lactate into the LECs. This was further supported by the lack of $^{13}\hbox {C}$-lactate uptake into LECs (Fig. 6b–d and S5).

While addition of lactate to the LEC medium caused significant changes in the overall metabolism of LECs, the results were different from similar studies in HUVECs^27^ and showed that lactate alone did not mediate the effects observed in co-culture. For example, co-culture led to increased lactate metabolism markers while lactate addition resulted in reduced lactate metabolism markers, potentially leading to a reduction of endogenous lactate production to reduce acidification of the medium. Lactate addition also led to increased redox ratio, possibly due to utilization of lactate to produce NAD(P)H via the TCA cycle and fatty acid oxidation. Other cell types, such as CAFs and non-small cell lung cancer cells have been shown to utilize lactate in a similar way^44–46^.

Interestingly, while $^{13}C$ NMR and 2D $^1\hbox {H}$–$^{13}\hbox {C}$ HSQC revealed it is unlikely that LECs in co-culture imported lactate secreted by breast cancer cells, LECs do uptake metabolites from breast cancer cells and provide a promising avenue of future research (Fig. 6 and S5).

Also, this work shows that lactate does not appear to influence lymphangiogenesis signaling in LECs (Fig. 7). Based on our data, LECs have a different response to high lactate concentrations compared to the reported response in HUVECs, where high lactate concentrations promoted angiogenic signaling^27–29,41^. Increased expression of lymphangiogenic markers was not due to increased lactate levels alone. In fact, high lactate present in the media seemed to have a suppressive effect of lymphangiogenic genes. The increase in lymphangiogenic signaling observed in LEC co-culture is likely due to paracrine signaling factors present in the co-culture conditions which aid LECs in adapting to higher lactate environments such as the acidic TME. Furthermore, increased tube formation was not observed despite increased lymphangiogenic gene expression. It is possible that the 2D tubulogenesis assay is not sensitive enough to detect the changes in lymphangiogenic signaling since LECs co-cultured with breast cancer cells in a microfluidic device did generate sprouting of new lymphatic vessels^18^. However, further research is needed to identify all the factors involved and their roles in LEC-breast cancer metabolic interactions.

## Materials and methods

### Cell maintenance and cell culture experiments

Breast cancer cells were purchased from the American Type Culture Collection (ATCC) and were cultured in high glucose Dulbecco’s Modified Eagle’s Medium (DMEM) supplemented with 10% FBS and 1% Penicillin-Streptomycin at $37\,^\circ \hbox {C}$ and 5% $CO_{2}$. MCF-7, MDA-MB-231 and SK-BR-3 cells were used to represent the different breast cancer subtypes and their characteristics are summarized in Table S1. LECs (Lonza) were cultured in endothelial cell growth media 2 microvascular (EGM2-MV; Lonza) at $37\,^\circ \hbox {C}$ and 5% $CO_{2}$. Experiments lasted 4 days and cell medium was replenished every 2 days for all experiments unless stated otherwise.

Breast cancer cells were co-cultured with endothelial cells using a 24 mm Transwell co-culture system; membrane pore size 0.4 $\upmu$m (Corning). LECs were seeded at a density of $7.5\,\times 10^{4}\,\hbox {cells/cm}^2$ and breast cancer cells were seeded at a density of $5.0\,\times 10^{4}\,\hbox {cells/cm}^2$. Cells were switched to EBM2 (Lonza) supplemented with 5% FBS during co-culture experiments. A co-culture control condition where the same cell type was cultured both on the Transwell membrane and bottom of the wells was employed to mimic total cell density conditions present in co-culture. This helped discern if observed metabolic changes were due to cancer-endothelial crosstalk or to an increase in cell density. For conditioned media experiments, breast cancer cells were cultured in EBM2 + 5% FBS for 2 days and this medium was collected. LECs were cultured for 4 days in 30% breast cancer conditioned medium. For lactate-spiked media experiments, LECs were cultured in EBM2 + 5% FBS + 10 mM sodium lactate (Sigma) medium. For $^{13}\hbox {C}$-labeled glucose experiments, breast cancer cells were cultured in DMEM containing no glucose supplemented with 10% FBS, 1% Penicillin-Streptomycin, and 25 mM $^{13}\hbox {C}$-glucose (Cambridge Laboratories Isotopes, Inc.) for 24 h. Then, co-culture with LECs was initiated as described before. LECs were analyzed by $^{13}\hbox {C}$ NMR 6 h after co-culture initiation.

### Metabolite extraction and $^1\hbox {H}$ NMR sample preparation

Adherent cells were incubated with 6 mL methanol for 2 min and mechanically detached using a cell scraper. The methanol/ cell mixture was collected and intracellular metabolites were isolated by using a 1:1:0.9 methanol/chloroform/water dual phase extraction procedure as described in^47^. The aqueous phase was collected for $^1\hbox {H}$ NMR spectroscopy and dried using a Vacufuge Plus (Eppendorf). Samples were reconstituted in 600 $\upmu$L of phosphate buffered deuterium oxide (${D_{2}}\hbox {O}$) solution [0.1 M ${D_{2}}\hbox {O}$ (Acros Organics), 0.5 mM 3-trimethylsilyl-propionate-2, 2, 3, 3,-d4 (TMSP; $\delta = 0.0$ ppm, internal standard), and 0.2% w/v sodium azide, pH 7.2]. Samples were spun at 18,000 g for 10 min and 550 $\upmu$L were transferred into 5 mm NMR tubes (Norell Inc.).

### NMR spectrum acquisition

All NMR spectra were analyzed at the National Magnetic Resonance Facility at Madison (NMRFAM) and acquired on a 500 MHz Bruker Avance III spectrometer with a 5 mm cryogenic probe at a temperature of 298 K. Line broadening was 0.5 Hz and the chemical shifts were referenced to the TMSP peak ($\delta =0.0$ ppm) using Bruker Top-Spin$^{TM}$ software (version 3.2.5).

1D $^1\hbox {H}$ NMR spectra were acquired using a nuclear Overhauser effect spectroscopy with presaturation and spoil gradients (NOESYGPPR1D) pulse sequence [relaxation delay = 2 s, mixing time = 10 ms, pre-scan delay = 30 $\upmu$s]. Each spectrum consisted of a spectral width of 12 ppm and 128 free induction decays (FIDs). For lactate-spiked media experiments, each spectrum contained 256 FIDs.

1D $^{13}\hbox {C}$ NMR spectra were acquired using zgpg30 pulse sequence [relaxation delay = 0.2 s and a pre-scan delay = 18 $\upmu$s]. Each spectrum consisted of 4096 FIDs and a spectral width of 236 ppm.

Two-dimensional $^1\hbox {H}$–$^{13}\hbox {C}$ HSQC spectra were acquired using hsqcetgpsisp2 pulse sequence. Each time-domain spectrum of the HSQC experiment was the average of 32 transients consisting of 4096 points; the second dimension was derived from 512 increments. The spectral widths were 12.5 ppm and 159 ppm for the $^1\hbox {H}$ and $^{13}\hbox {C}$ dimensions, respectively.

### Data processing and multivariate statistical analysis for metabolomics

Data was processed and analyzed as described in^48^. Briefly, phase and baseline corrections and water region removal (4.7–5 ppm) were performed using ACD/1D NMR Processor software (Advanced Chemistry Development). ChenomX NMR Suite Profiler (version 7.7, ChenomX Inc.) was utilized to determine metabolite concentration using TMSP as a reference compound. To ensure that highly variable metabolites would not influence metabolomics analysis, the coefficient of variation (CV) was calculated by dividing the standard deviation by the sample mean for all features. Metabolites with high CV values (CV > 0.30) were excluded from analysis. Exceptions were made for low-concentration metabolites with consistent concentration values between replicates given that in these cases any small change in concentration would generate a high CV value.

Metabolite concentration data were imported into Metaboanalyst^33,49–51^ and normalized by the total concentration for each sample and scaled using auto-scaling. PCA and hierarchical clustering using Pearson correlation and Ward clustering were performed on the metabolite concentration data using the Statistics module. Metabolites identified as significant by one-way ANOVA followed by a Tukey’s HSD post-hoc analysis (to determine which comparisons between the five groups were significant; FDR < 0.05) [calculated using the Statistics module in Metaboanalyst] were log-transformed and graphed in GraphPad Prism software (Graphpad Prism 7.04). Differentially enriched metabolic pathways obtained from quantitative MSEA^52^, performed using the Enrichment module in Metaboanalyst, were visually represented as networks using the Metscape 3 plug-in in Cytoscape^53,54^ or heatmaps using Morpheus software^55^.

The metabolomics data reported in this paper are available in the MetaboLights database, study identifier MTBLS859.

### Quantitative RT-PCR analysis

Total RNA from cells was isolated and purified using the PureLink RNA Mini Kit (Life Technologies). RNA concentration was determined using a NanoDrop$^{\mathrm{TM}}$ 2000/c (Thermo Fisher Scientific). For co-culture samples, 285 $\upmu$g of RNA were analyzed and for lactate-spiked media samples, 55 $\upmu$g of RNA were used. cDNA synthesis was performed using the SuperScript III First-Strand Synthesis SuperMix for qRT-PCR kit (Invitrogen).

1 $\upmu$L of cDNA per sample was analyzed by qRT-PCR using nuclease-free water, a 1:1 mix of forward: reverse primers at 10 $\upmu$M concentration, and Power SYBR Green PCR Master Mix (Applied Biosystems). The samples were analyzed using an AriaMX Real-Time PCR System (Agilent Techologies) using the following thermal profile: a hot start of $95\,^\circ \hbox {C}$ for 15 min, a cycle of $95\,^\circ \hbox {C}$ for 15 s and $60\,^\circ \hbox {C}$ for 1 min repeated for 45 cycles, and a melting curve of 95–60 $^\circ \hbox {C}$ with $0.5\,^\circ \hbox {C}$ intervals. Primer sequences (purchased from Integrated DNA Technology [IDT]) are in Table S2.

### Western blot analysis

Cold RIPA buffer (Thermo Scientific) + 1$\times$ Halt protease inhibitor cocktail (Thermo Scientific) was added to LECs and incubated at $4\,^\circ \hbox {C}$ for 15 min. Cells were mechanically harvested using a cell scraper and centrifuged at 18,000*g* for 5 min at $4\,^\circ \hbox {C}$. Protein concentration was determined using a bicinchoninic acid assay (BCA) Assay (Thermo Fisher Scientific) following manufacturer’s directions. Protein samples, composed of 3$\times$ polyacrylamide gel electrophoresis (PAGE) Sample Buffer, water, and protein lysate were prepared and heated at $95\,^\circ \hbox {C}$ for 5 min. The PAGE buffer consisted of Tris base, sucrose, bromophenol blue, 20% sodium dodecyl sulfate (SDS) in water, $\beta$-mercaptoethanol, and water. Subsequently, 30 $\upmu$L of sample was loaded onto 4–12% Tris-Glycine Mini Gels (Invitrogen). SeeBlue Plus2 Pre-stained Standard (Life Technologies) and MagicMark$^{\mathrm{TM}}$ XP Western Standard (Invitrogen) were used as protein ladders. Gels were run at 125 V for 75 min. Protein samples were then transferred onto a nitrocellulose membrane at 100 V for 1 h on ice.

For antibody labeling, membranes were incubated in 5% non-fat milk or 5% bovine serum albumin (BSA) in 1$\times$ TBST for 60 min and subsequently incubated with primary antibody in 5% non-fat milk or 5% BSA in TBST overnight at $4\,^\circ \hbox {C}$. Antibodies used are summarized in Table S3. Membranes were washed three times with TBST for 15 min followed by a 5 min TBST wash. Secondary antibodies (Li-Cor) were added to membranes and incubated at room temperature for 60 min. After washing the membranes with TBST, these were imaged using the Odyssey Classic Imager (Li-Cor).

Images were exported at a resolution of 300 dpi and band intensity was determined using ImageJ for protein quantification measurements. LDHA, LDHB, and MCT4 protein expression were normalized to $\beta$-actin. For display images, the background was subtracted and the signal was sharpened using ImageJ.

### Lactate secretion quantification

Lactate present in the media was quantified using the L-Lactate Assay Kit (Abcam) following the manufacturer’s instructions. Media samples were de-proteinized prior to lactate secretion quantification by centrifuging samples using 10 kDa $\hbox {Corning}^{\mathrm{TM}}$ Spin-$\hbox {X}^{\mathrm{TM}}$ UF Concentrators (Corning) at manufacturer’s suggested speed for 5 min.

### Optical redox ratio imaging

Fluorescence imaging was performed using a custom-built multiphoton microscope (Bruker Fluorescence Microscopy, Middleton, WI). Illumination was provided with a pulsed Ti:Saph laser (Insight DS+, Spectra Physics, Santa Clara, CA), tuned to 750 nm for NAD(P)H excitation, and 890 nm for FAD excitation. Excitation and emission were coupled using a 40X water-immersion objective (Nikon, 1.15 NA). Fluorescence emission of NAD(P)H was isolated with a 440/80 nm bandpass filter cube, and fluorescence emission from FAD was isolated using a 550/100 nm filter cube. Images were acquired with a resolution of 256 x 256 px, with an optical zoom of 1.16 and a pixel dwell time of 4.8 $\upmu$s over a 60 s integration time. A minimum of 6 fields-of-view were collected for each treatment.

### Redox ratio quantification

Images were analyzed using Cell Profiler software. Images were thresholded based on intensity, and masks were used to isolate only cells as regions of interest. A redox ratio was calculated for each image by dividing the unmasked NAD(P)H fluorescence intensity by the unmasked FAD fluorescence intensity. Average redox ratios for each treatment group were calculated and normalized to LEC cc or 0 mM conditions as appropriate.

### Seahorse XF96 assay

LECs were co-cultured with LECs (LEC cc; control) or cancer cells (MCF-7, MDA-MB-231, and SK-BR-3) for 4 days prior to assay as described above. Further, a LEC cc supplemented with 10mM L-Lactic acid for 4 days was performed as well. On day 4, LECs were lifted, seeded at 40k cells per well of a seahorse XF96 cell culture micro-plate in 100 $\upmu$L 50:50 conditioned: fresh media, and allowed to attach for 5 h. Wells were rinsed 3 times with XF RPMI1640 Base Media supplemented with 5.5 mM glucose, 1 mM pyruvate, 2 mM glutamine (pH 7.4), followed by addition to a final volume of 180 $\upmu$L of media. The Cell Energy Phenotype Test (Agilent Technologies) was performed per manufacturer’s instruction with the Wave software (Agilent Technologies). In brief, after equilibration 3 baseline oxygen consumption rate (OCR) and extracellular acidification rate (ECAR) measurements were acquired followed by injection of oligomycin and FCCP (1 uM final concentration each) and 5 additional measurement acquisitions. The resulting data was processed with the Seahorse XF Cell Energy Phenotype Test Report Generator (Agilent Technologies).

### Cord network formation assay

24-well plates were coated with 300 $\upmu$g of 8 $\upmu$g/mL Matrigel (WiCell). Endothelial cells were seeded on the Matrigel coating at a concentration of $2\times 10^{5}$ cells/well and left to attach for 1 h. Breast cancer cells were seeded on the top membrane of a 24 mm Transwell (Corning) at a density of $5\times 10^{4}\,\hbox {cells/cm}^{2}$. Co-culture was initiated after 1 h. Cells were imaged using a Nikon microscope 10 h after co-culture initiation. Brightfield images of a $2 \times 2$ grid in each well were acquired using a 10x objective. The Angiogenesis Analyzer plug-in in Image J/Fiji software was used to analyze the brightfield images and quantify junctions per area, branching per area and average tube length^56^.

## Supplementary information

Supplementary information.
