# Supplementary material for: Metabolomics revealed the influence of breast cancer on lymphatic endothelial cell metabolism, metabolic crosstalk, and lymphangiogenic signaling in co-culture
Source: Sci Rep. 2020 Dec 4;10:21244. doi: 10.1038/s41598-020-76394-7 (PMC7718899; doi:10.1038/s41598-020-76394-7)
Supplement: Supplementary file 1 — Supplementary information. [file 41598_2020_76394_MOESM_ESM.pdf]

Metabolomics revealed the influence of breast cancer on lymphatic endothelial cell metabolism, metabolic crosstalk, and lymphangiogenic signaling in co-culture.

Suehelay Acevedo-Acevedo[1,*], Douglas C. Millar[2], Aaron D. Simmons[2], Peter Favreau[3], Paulo F. Cobra[4], Melissa Skala[1,3], and Sean P. Palecek[1,2]

[1]University of Wisconsin-Madison, Department of Biomedical Engineering, Madison, WI, 53706, USA
[2]University of Wisconsin-Madison, Department of Chemical and Biological Engineering, Madison, WI, 53706, USA
[3]Morgridge Institute for Research, Madison, WI, USA
[4]University of Wisconsin-Madison, Department of Biochemistry, Madison, WI, 53706, USA
[*]Corresponding author: Suehelay.AcevedoAcevedo@moffitt.org.

S1 Supplementary Figures and Tables

Table S1. Description of breast cancer cell lines co-cultured with LECs. Adapted from[1,2].

Cell line	MCF7	MDAMB231	SKBR3
Subtype	Luminal A	Claudin-low/ Basal-like	HER2
Receptor	ER+, PR+, HER2-	ER-, PR-, HER2-	ER-, PR-, HER2+
Invasive	Low	High	High

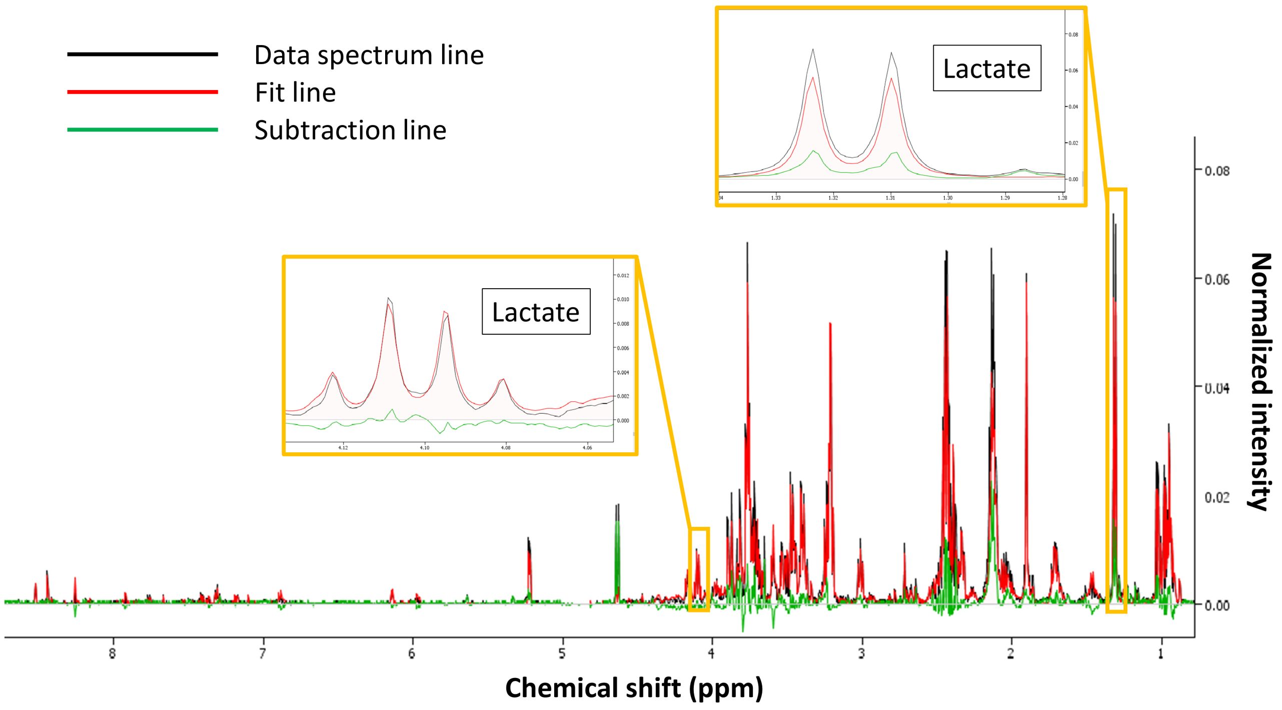

Figure S1. Representative ^{1}H NMR spectral fitting using ChenomX Software to determine metabolite concentrations. One LEC cc replicate is shown as an example. The black data spectrum line is the original NMR data signal, the red fit line is the software metabolite fit, and the green subtraction line is the difference between the black and red lines.

Table S2. Primer sequences used for qRT-PCR.

Gene	Forward primer sequence	Reverse primer sequence	NCBI accession number
FLT4	5'-CATCTACAAAGACCCCGACTAC-3'	5'-CCAGAGAAGCACCCCAAAG-3'	NM_002020
GAPDH	5'-GAAGGTGAAGGTCGGAGTCAACG-3'	5'-TCCTGGAAGATGGTGATGGGAT-3'	NM_002046.5
HIF1A	5'-AACATAAAGTCTGCAACATGGAAG-3'	5'-TTTGATGGGTGAGGAATGGG-3'	NM_001530
IL8	5'-CGGAAGGAACCATCTCACTG-3'	5'-ACTGCACCTTCACACAGAGC-3'	NM_000584.3
LDHA	5'-AGATTCCAGTGTGCCTGTATG-3'	5'-CCAATAGCCCAGGATGTGTAG-3'	NM_001135239
LDHB	5'-CACCAGTTGCGGAAGAAGAG-3'	5'-CAGCCAGAGACTTTCCCAGA-3'	NM_001174097.2
MCT1 (SLC16A1)	5'-CTGTCATGTATGGTGGAGGTC-3'	5'-GGTCAGAGCTGGATTCAAGTTG-3'	NM_003051
MCT4 (SLC16A3)	5'-CCATGCTCTACGGGACAG-3'	5'-GAGGGCTGGAAGTTGAGTG-3'	NM_001042422
NFKB1	5'-ACCCTGACCTTGCCTATTTG-3'	5'-GAAAAGCTGTAAACATGAGCCG-3'	NM_003998
VEGFC	5'-ACAAGTGTCAGCTAAGGAAAGG-3'	5'-CCAAACTCCTTCCCCACATC-3'	NM_005429

Table S3. Antibodies used for western blot analysis.

Protein	Clonality	Host	Concentration	Company
β-actin (13E5)	Monoclonal	Rabbit	1:1000	Cell Signaling Technologies
LDHA (C4B5)	Monoclonal	Rabbit	1:1000	Cell Signaling Technologies
LDHB (2057D)	Monoclonal	Rabbit	1:1000	R & D Systems
MCT4	Polyclonal	Rabbit	1:1000	EMD Millipore
IRDye 800 CW anti-rabbit	Polyclonal	Goat	1:5000	Li-Cor

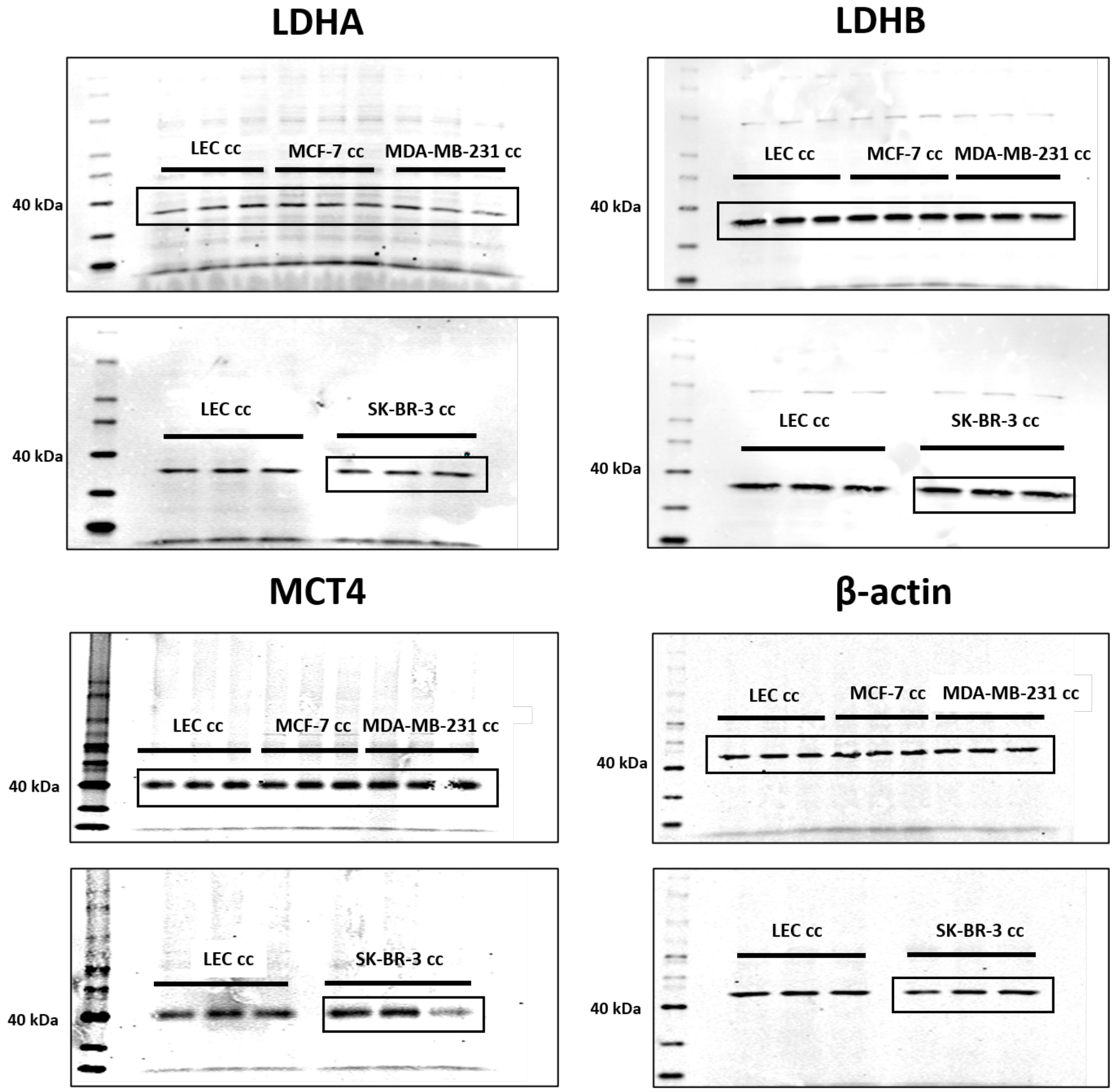

Figure S2. Full western blot images from LECs co-cultured with breast cancer cells. Blots were exposed to 800 nm light at an intensity setting of 7 and were imaged using the Odyssey Classic Imager (Li-Cor). Black outlines depict the regions of the blots that were cropped to generate the representative image in Fig 5. For display images, the background was subtracted, and the signal was sharpened using ImageJ.

(a) (b)

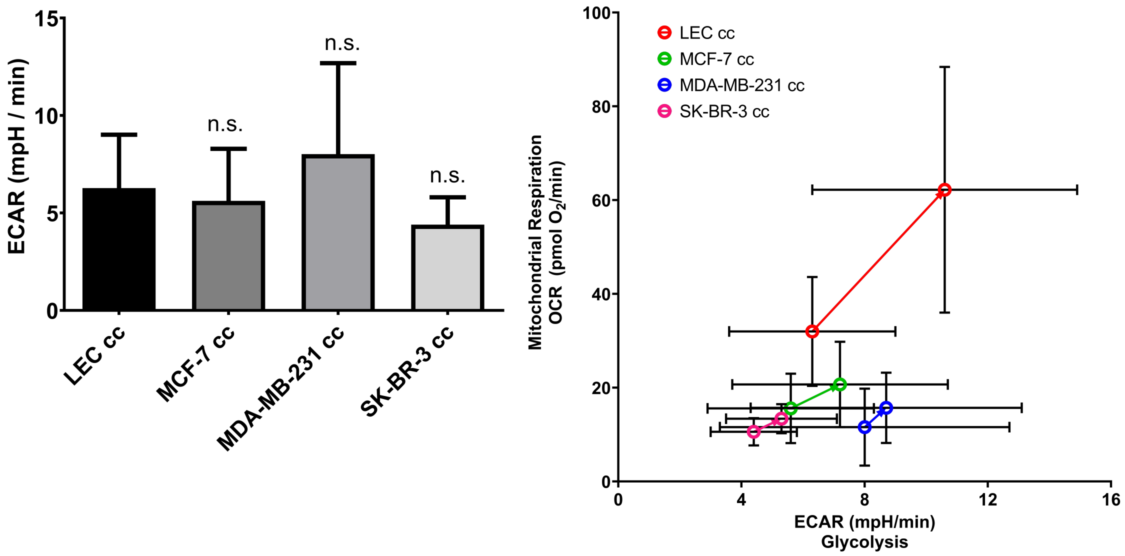

Figure S3. (a) Baseline extracellular acidification rate (ECAR) measurements of LECs co-cultured with breast cancer cells. Statistics were calculated using one-way ANOVA with Sidak's multiple comparisons test (GraphPad Prism 8.03). N= 12- 17 biological replicates; n.s. = not significant. (b) Summary of Seahorse Cell Energy Phenotype analysis on LEC cc (red), MCF-7 cc (green), MDA-MB-231 cc (blue), and SK-BR-3 cc (pink). Error bars represent standard deviation.

(a) (b)

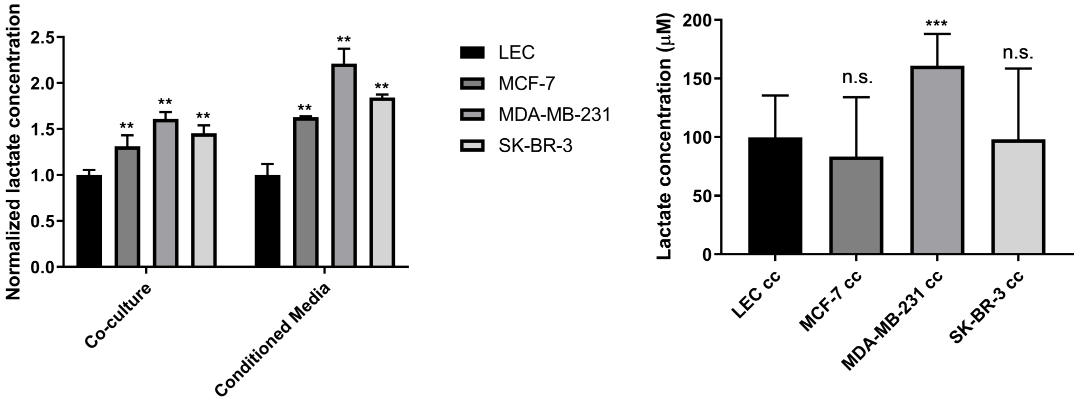

Figure S4. Co-culture with breast cancer cells significantly increased intracellular lactate concentrations in LECs and lactate concentration in the media. (a) Intracellular lactate was quantified using ^{1}H NMR spectroscopy for LECs that were cultured with the indicated breast cancer cells or in breast cancer cell-conditioned media for 4 days. Significance relative to LEC cc control was determined by one-way ANOVA with Tukey's HSD post-hoc analysis. **FDR $<$ 0.05. n = 3. (b) Lactate levels in cell culture media were quantified for LECs in co-culture with the indicated breast cancer cells. Statistics were calculated using an unpaired t-test (GraphPad Prism 7.04). ***p-value $<$ 0.0005. n = 9; 3 independent experiments. n.s. = not significant (p-value $>$ 0.05). Error bars represent standard deviation.

(a)

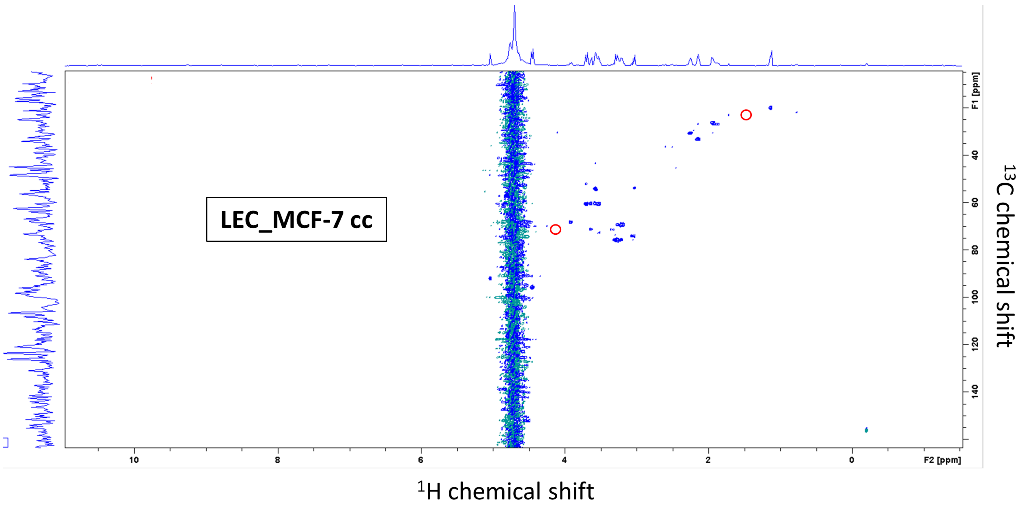

(b)

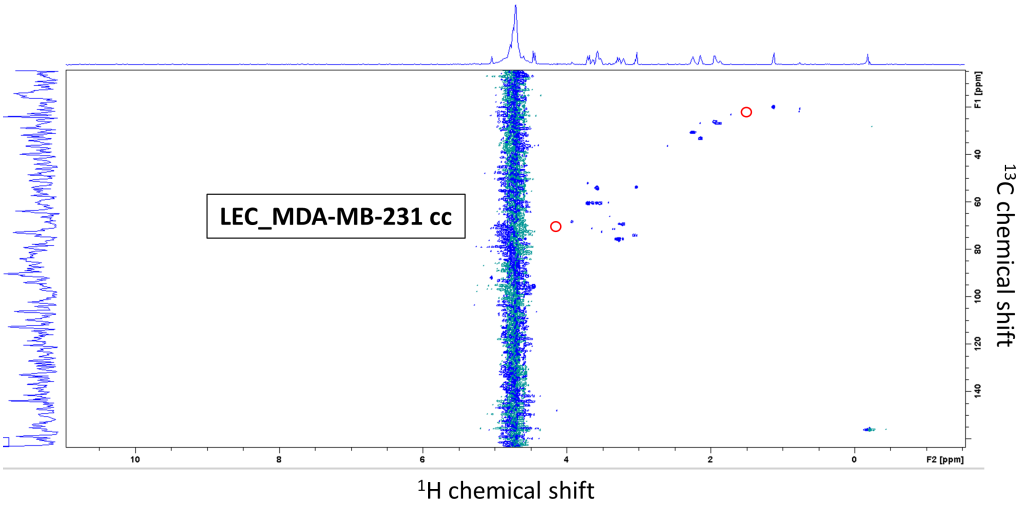

(c)

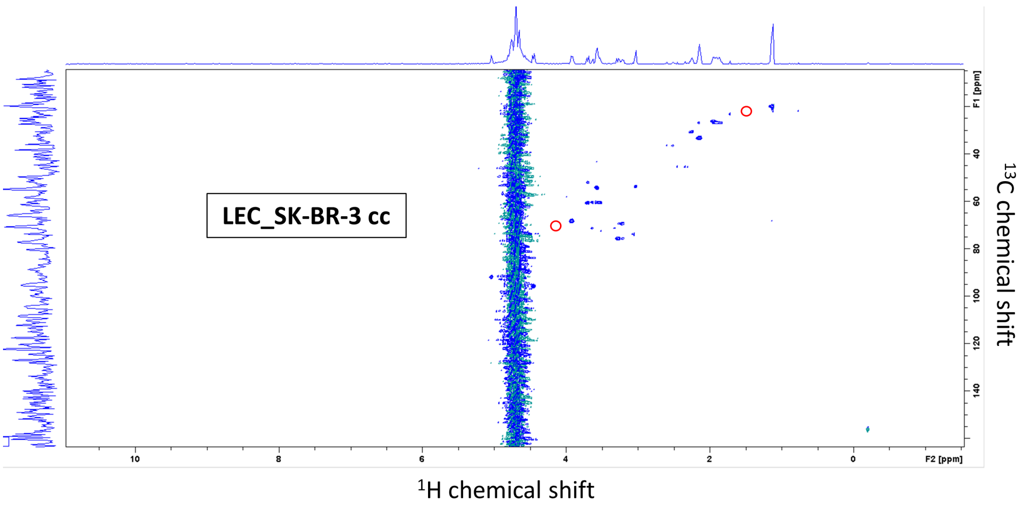

Figure S5. 2D ^{1}H-^{13}C HSQC NMR spectra of LECs co-cultured with MCF7 (a), MDAMB231 (b), and SKBR3 (c) cells showed no lactate present inside LECs. Red circles indicate the expected location of the signal corresponding to ^{13}C-lactate.

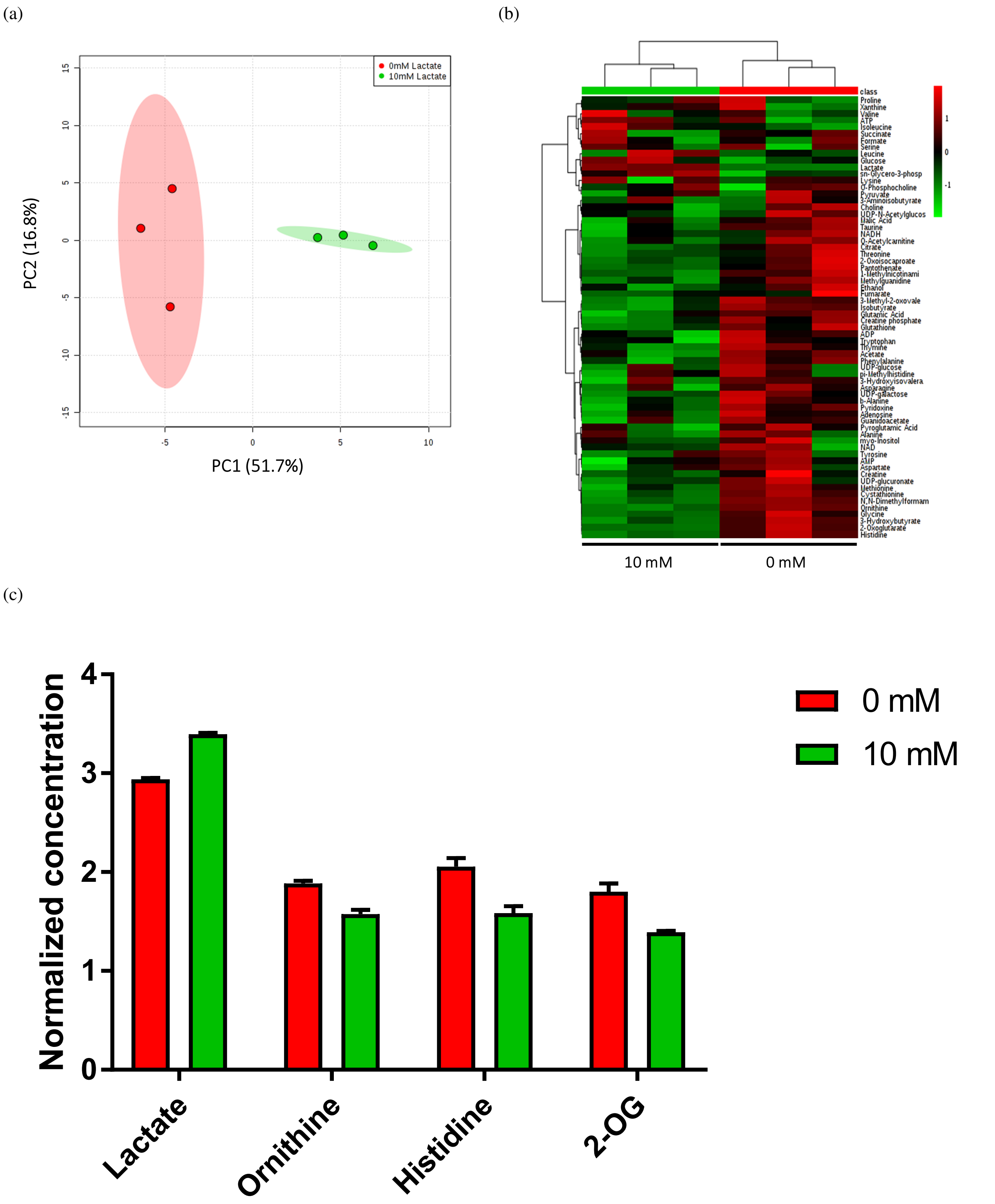

Figure S6. High lactate media conditions elicited differences in LEC metabolism. (a) PCA score plot of LECs cultured in 10 mM lactate (green) and medium containing no lactate (red). (b) Hierarchical clustering was performed using Pearson's correlation as a distance measure and Ward's algorithm for clustering. (c) Significantly changing metabolites between 0 mM and 10 mM conditions were determined by Volcano plot analysis; fold change > 2; p-value < 0.05. n = 3 samples. Analyzed using Metaboanalyst[3].

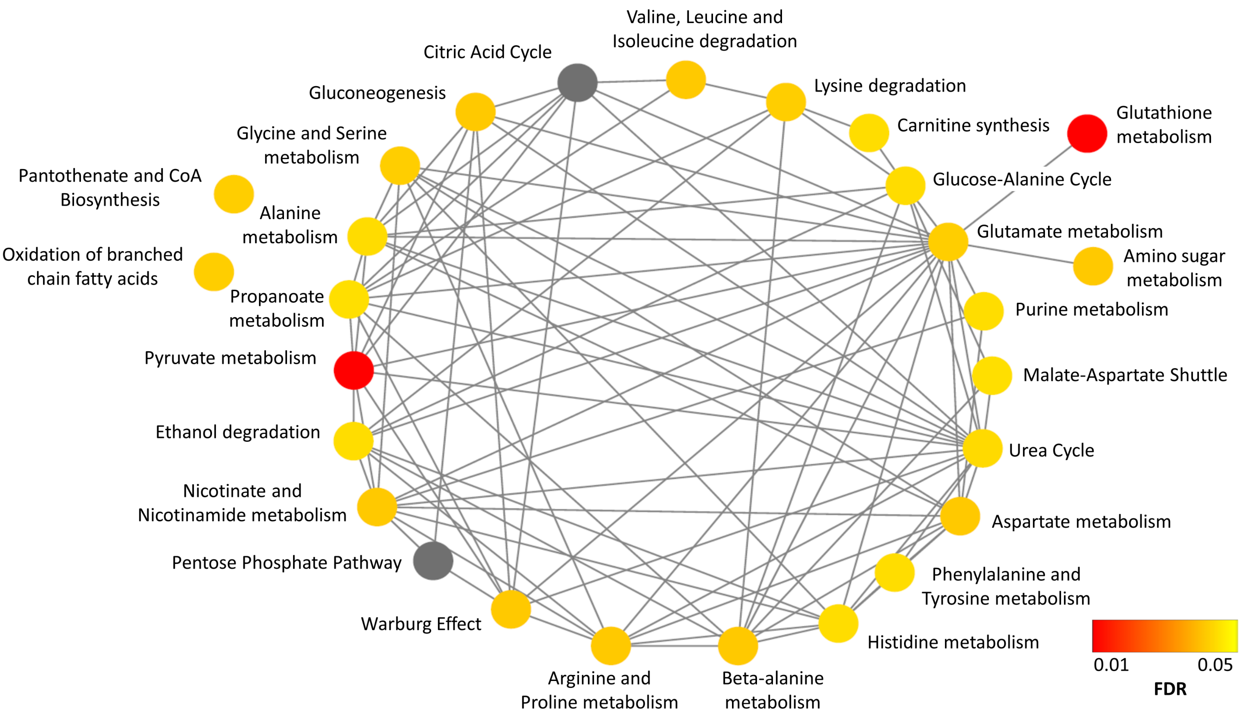

Figure S7. 25 metabolic pathways were enriched in LECs cultured in high lactate medium. MSEA of metabolite concentration data from LECs cultured with or without spiking the medium with lactate. Each node was colored according to the FDR values determined by one-way ANOVA. Grey nodes indicate no significant enrichment. n = 3 biological replicates. Analyzed using Metaboanalyst[3] and network generated using Metscape.

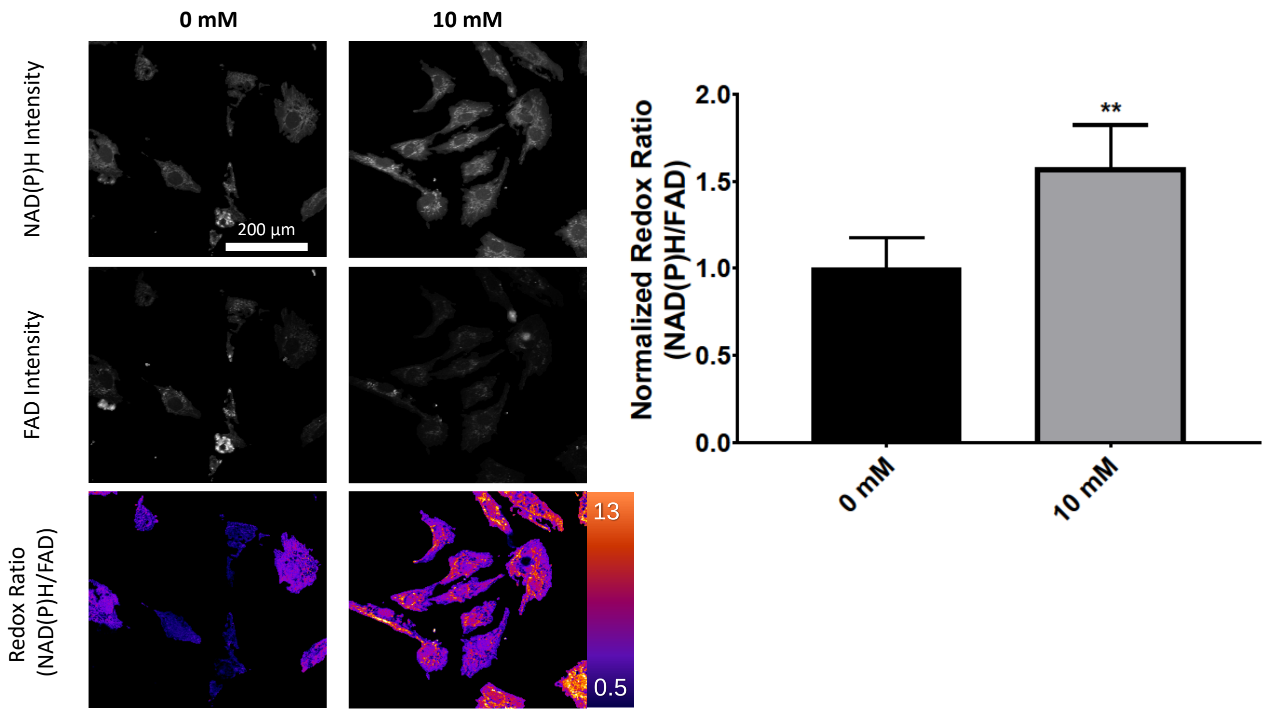

Figure S8. Representative intensity and optical redox ratio images from LECs cultured in control medium or medium supplemented with 10 mM lactate. Error bars represent standard deviation. Statistics were calculated using a Mann-Whitney U test (GraphPad Prism 7.04). ** $p < 0.005$

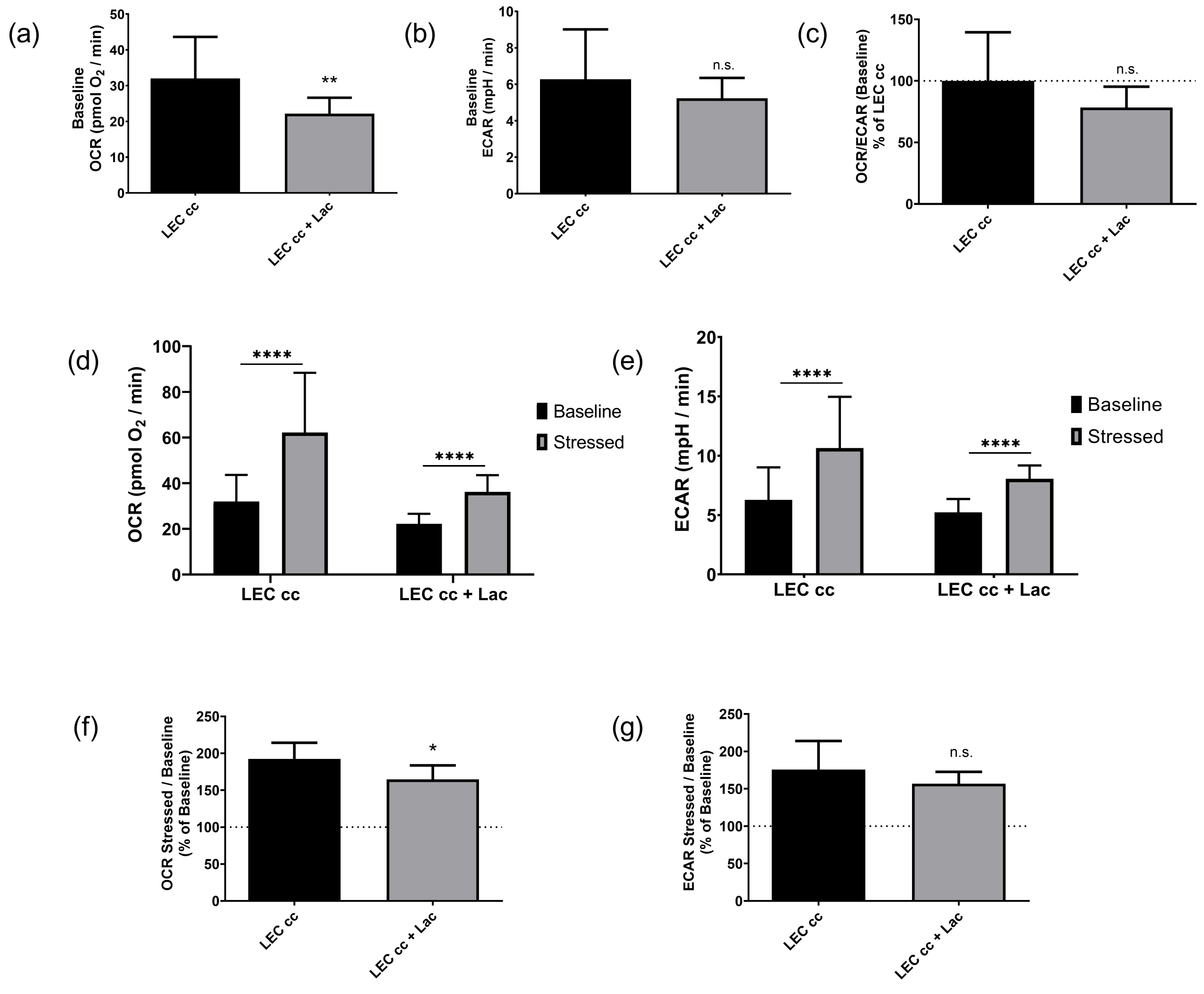

Figure S9. High lactate did not affect the metabolic response of LECs to mitochondrial stressor compounds and increased energy demand. (a) Baseline oxygen consumption rate (OCR) measurements of LEC cc and LEC cc supplemented with 10 mM lactate (LEC cc + lac). (b) Baseline extracellular acidification rate (ECAR) measurements of LEC cc and LEC cc supplemented with 10 mM lactate (LEC cc + lac). (c) Baseline OCR: ECAR ratio graphed as % of LEC cc. (d) OCR and (e) ECAR were measured at baseline and after oligomycin and FCCP addition (stressed condition). The ratio of stressed to baseline response was calculated for (f) OCR and (g) ECAR measurements. Statistics were calculated using one-way ANOVA with Sidak's multiple comparisons test (GraphPad Prism 8.03). *p-value < 0.05; **p-value < 0.005; ***p-value < 0.0005; ****p-value < 0.0001; n.s. = not significant (p-value > 0.05). n = 12- 17 biological replicates. Error bars represent standard deviation.

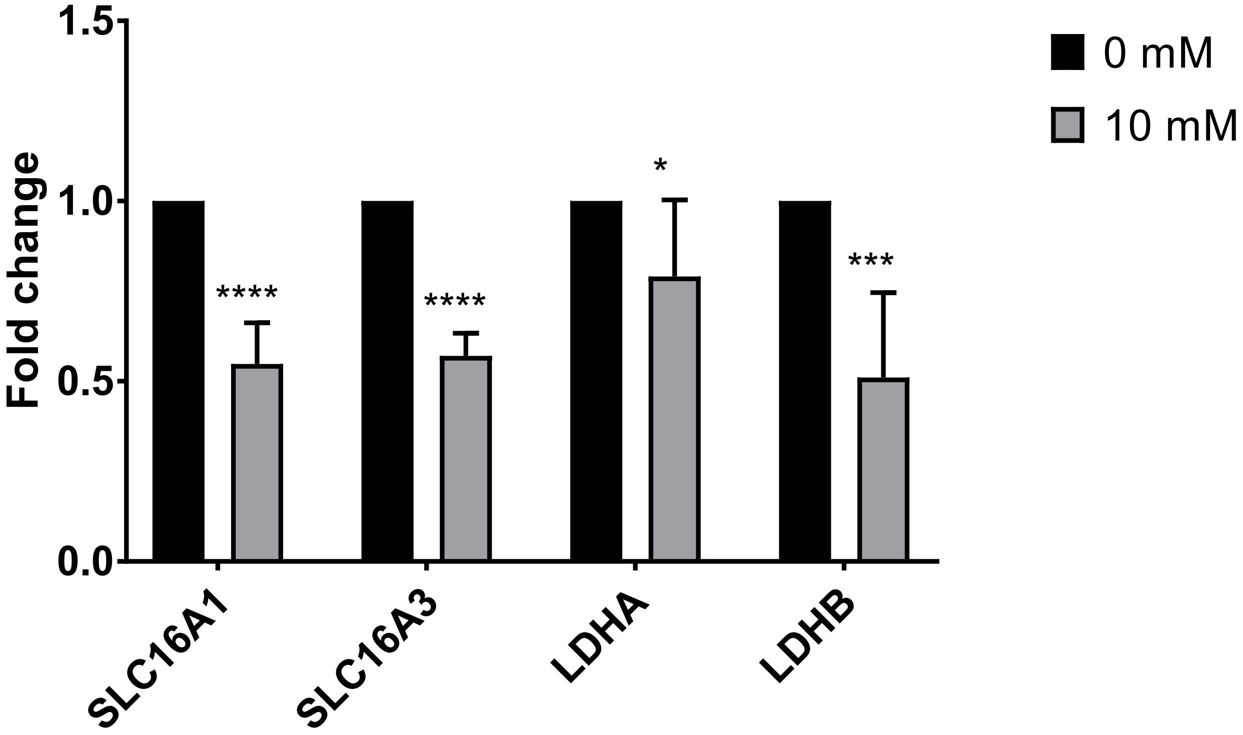

Figure S10. Genes involved in lactate metabolism and transport were significantly downregulated in LECs cultured in high lactate conditions. qRT-PCR gene expression measured in LECs cultured in EBM2 + 5% FBS medium spiked with 10 mM lactate or no lactate (control) was normalized to *GAPDH* gene expression. T-test with twp-step Benjamini, Krieger and Yekutieli procedure was performed to determine significant changes compared to LEC cc (GraphPad Prism 7.04). *p-value < 0.05; ***p-value < 0.001; ****p-value < 0.00001. Error bars represent standard deviation.

(a)

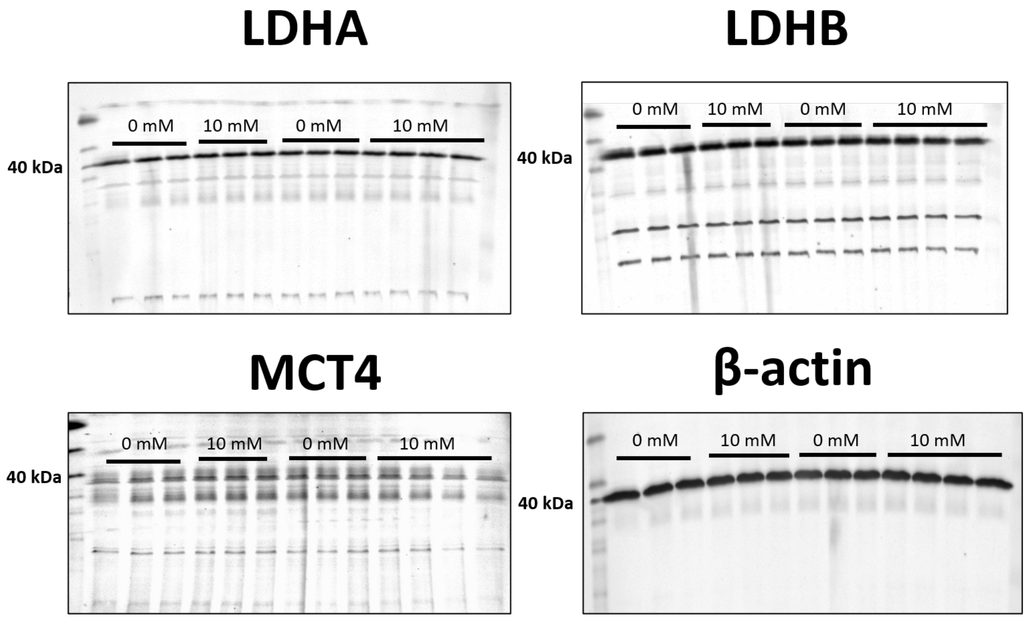

(b)

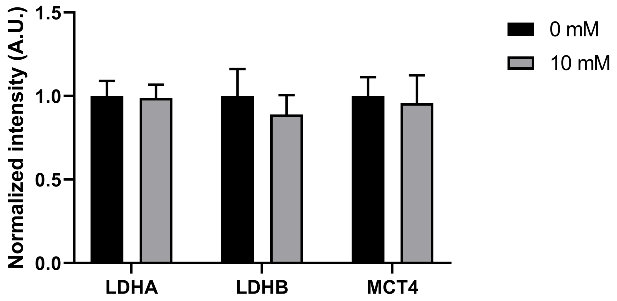

Figure S11. Proteins associated with lactate metabolism and transport showed no change in LECs cultured in high lactate conditions. (a) Representative images of western blots for LDHA, LDHB, and MCT4 protein expression in LECs. For display images, the background was subtracted, and the signal was sharpened using ImageJ. Band intensity values were normalized to β-actin expression and graphed in (b). Data was not statistically significant as determined by t-test with two-step Benjamini, Krieger and Yekutieli procedure (GraphPad Prism 7.04). Error bars represent standard deviation. n = 3 biological replicates.

(a)

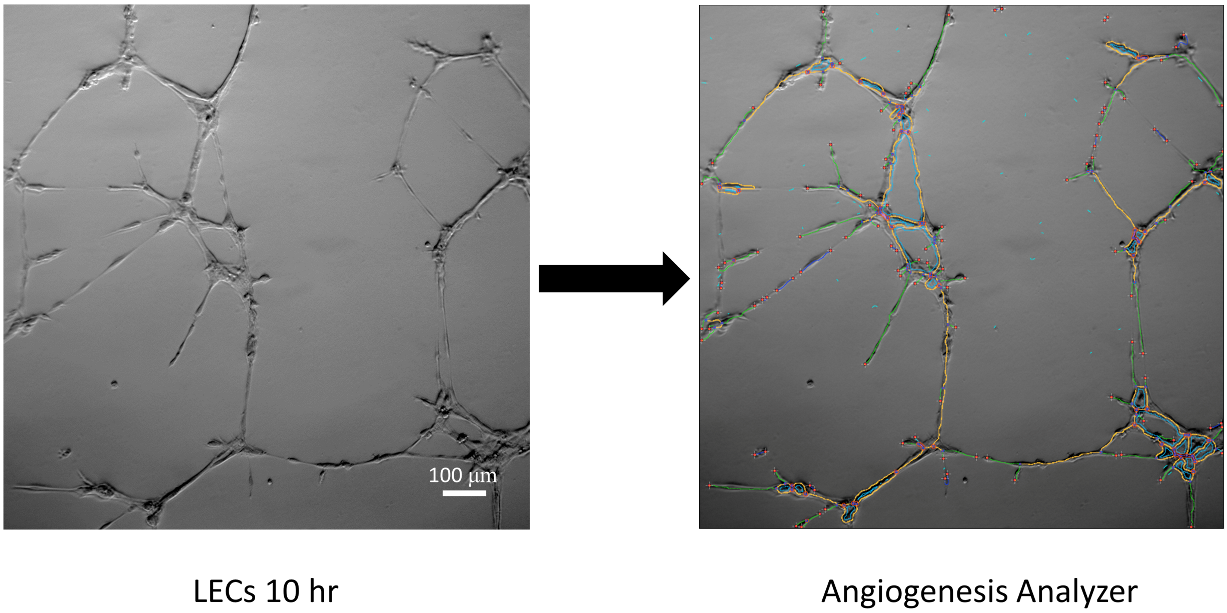

(b)

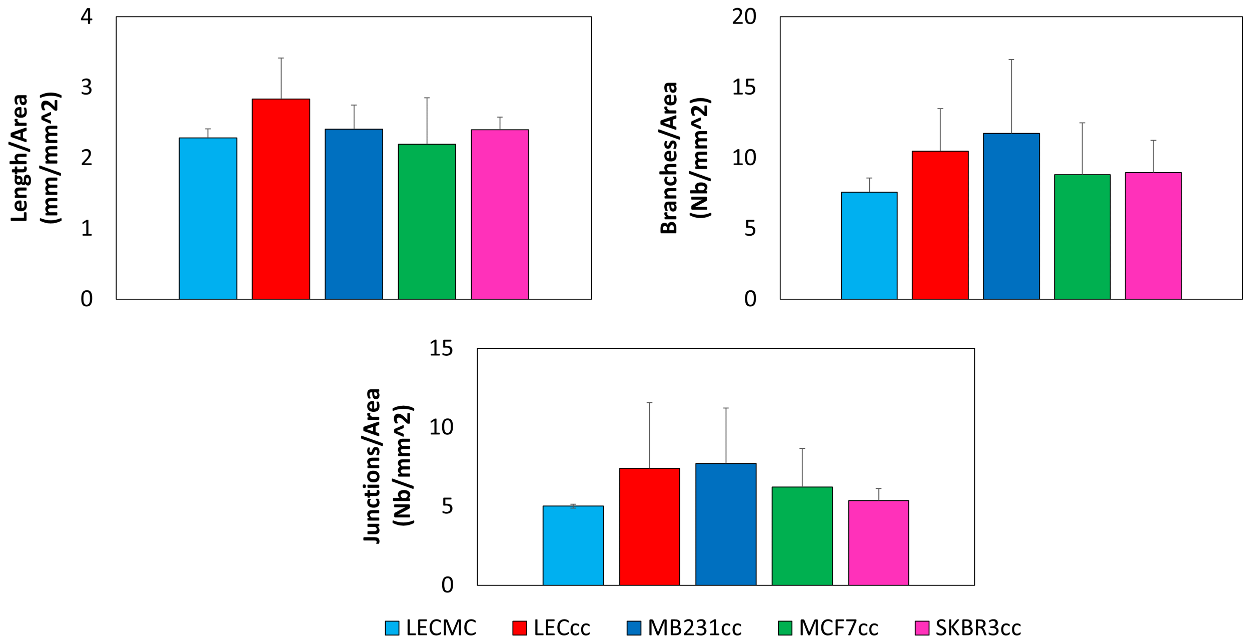

Figure S12. Co-culture with breast cancer cells did not impact LEC tubulogenesis. (a) Representative image of cord formation of LECs before and after analysis using the Angiogenesis Analyzer software. (b) Cord length, branches and junctions for LECs co-cultured with breast cancer were quantified 10 hours after co-culture initiation. Images were normalized by image area. Data was not statistically significant as determined by unpaired t-test (GraphPad Prism 7.04). n = 9 biological replicates, 3 independent experiments. Error bars represent standard deviation.

References

1. Neve, R. M. *et al.* A collection of breast cancer cell lines for the study of functionally distinct cancer subtypes. *Cancer Cell* **10**, 515–527, DOI: 10.1016/j.ccr.2006.10.008 (2006).

2. Holliday, D. L. & Speirs, V. Choosing the right cell line for breast cancer research. *Breast Cancer Res.* **13**, 215, DOI: 10.1186/bcr2889 (2011).

3. Xia, J., Psychogios, N., Young, N. & Wishart, D. S. MetaboAnalyst: a web server for metabolomic data analysis and interpretation. *Nucleic Acids Res.* **37**, W652–W660, DOI: 10.1093/nar/gkp356 (2009).
